# WetA bridges cellular and chemical development in *Aspergillus flavus*

**DOI:** 10.1371/journal.pone.0179571

**Published:** 2017-06-28

**Authors:** Ming-Yueh Wu, Matthew E. Mead, Sun-Chang Kim, Antonis Rokas, Jae-Hyuk Yu

**Affiliations:** 1 Departments of Bacteriology and Genetics, University of Wisconsin, Madison, Wisconsin, United States of America; 2 Department of Biological Sciences, Vanderbilt University, Nashville, Tennessee, United States of America; 3 Department of Biological Sciences, Korea Advanced Institute of Science and Technology, Dae-Jon, Republic of Korea; University of Nebraska-Lincoln, UNITED STATES

## Abstract

Bridging cellular reproduction and survival is essential for all life forms. *Aspergillus* fungi primarily reproduce by forming asexual spores called conidia, whose formation and maturation is governed by the central genetic regulatory circuit BrlA→AbaA→WetA. Here, we report that WetA is a multi-functional regulator that couples spore differentiation and survival, and governs proper chemical development in *Aspergillus flavus*. The deletion of *wetA* results in the formation of conidia with defective cell walls and no intra-cellular trehalose, leading to reduced stress tolerance, a rapid loss of viability, and disintegration of spores. WetA is also required for normal vegetative growth, hyphal branching, and production of aflatoxins. Targeted and genome-wide expression analyses reveal that WetA exerts feedback control of *brlA* and that 5,700 genes show altered mRNA levels in the mutant conidia. Functional category analyses of differentially expressed genes in Δ*wetA* RNA-seq data indicate that WetA contributes to spore integrity and maturity by properly regulating the metabolic pathways of trehalose, chitin, α-(1,3)-glucan, β-(1,3)-glucan, melanin, hydrophobins, and secondary metabolism more generally. Moreover, 160 genes predicted to encode transcription factors are differentially expressed by the absence of *wetA*, suggesting that WetA may play a global regulatory role in conidial development. Collectively, we present a comprehensive model for developmental control that bridges spore differentiation and survival in *A*. *flavus*.

## Introduction

Coordination of cellular reproduction and survival is fundamental to the existence and propagation of all living organisms. From the simplest single cell organisms to complex multicellular plants and animals, regulatory and signalling systems have evolved to ensure that future viability of the reproductive cells. Fungi primarily reproduce through spore propagation; fungal spores are adapted for dispersal and are resistant to desiccation, heat, oxidative and UV stresses, properties which also render them very capable of establishing infections [1]. Fungal sporulation involves coordinated control of morphological, physiological, and metabolic (chemical) developmental processes.

The genus *Aspergillus* includes several organisms that are commonly found in the human environment. For example, the widely distributed *Aspergillus flavus* is an opportunistic pathogen of plants and humans [2], and can produce the mycotoxin aflatoxin B1 (AFB1), the most potent carcinogen found in nature. The main means of dissemination of this fungus is producing a massive number of asexual spores (conidia), which are dispersed in the soil and air. In agricultural fields, these spores are carried to corn ears by insects or the wind where they grow in maize kernels and produce AFB1 [3]. AFB1 can be present in oil-seed crops, such as corn, cereals, sorghum, and peanuts, and when AFB1 is present in the feed consumed by a cow, it can be metabolised to AFM1 (M for milk), which is also highly toxic and carcinogenic [4]. Consumption of high doses of AFB1 in humans can lead to acute aflatoxicosis, liver necrosis, and even death. Due to their carcinogenicity and toxicity, levels of aflatoxins in foods and feeds are strictly regulated worldwide [5]. As conidiation and AF production are tightly correlated in *A*. *flavus*, understanding the mechanisms bridging cellular and chemical development may provide novel insights into controlling the dissemination of the fungus and subsequent contamination of crops by AFB1 [6–8].

**Fig 1:**
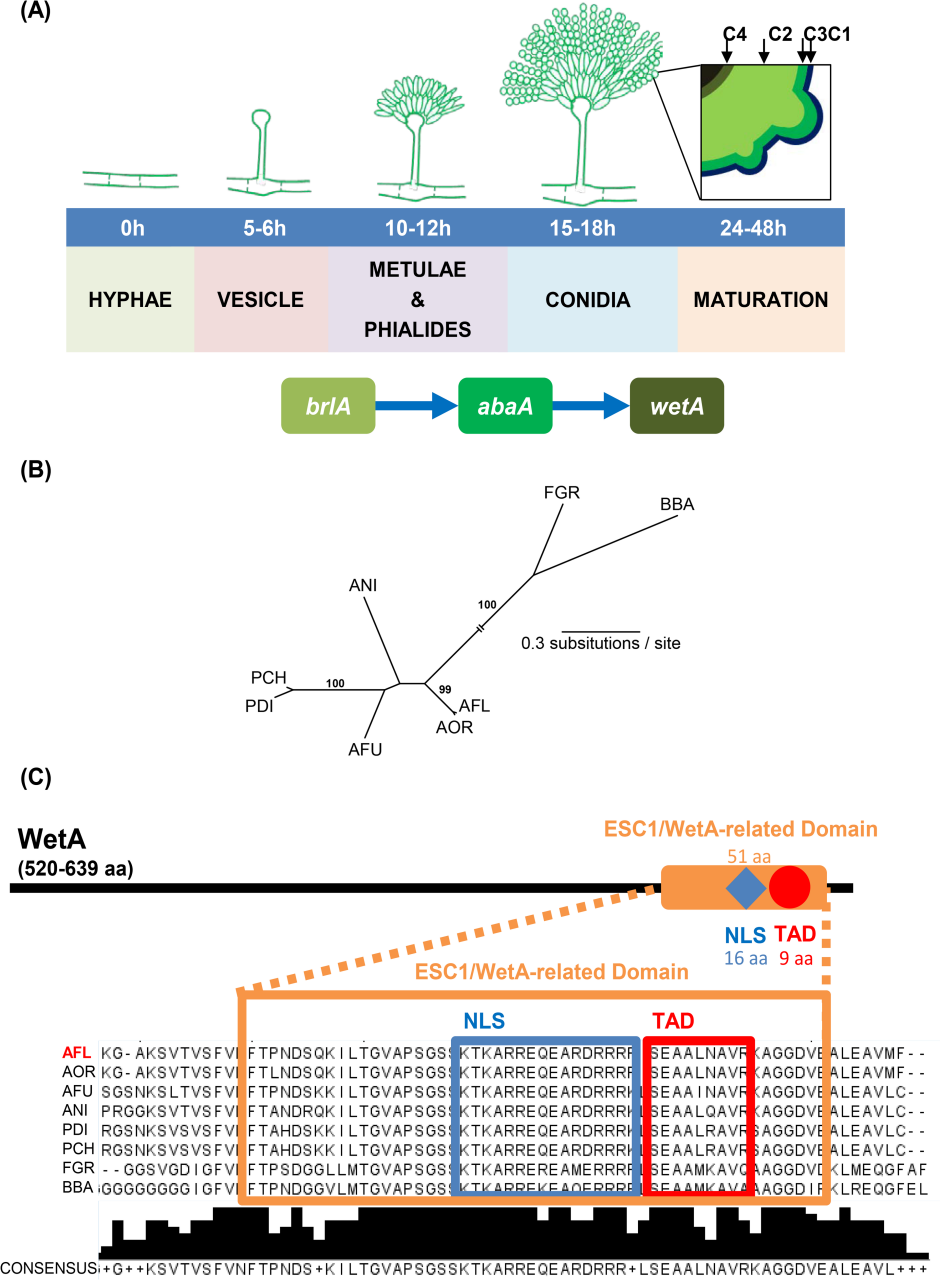
WetA is required for proper conidial maturation and contains both a transcription activation domain and a nuclear localization signal in a variety of fungi. (A) A model for the roles of the central regulators in *Aspergillus* conidiogenesis. WetA is activated by AbaA and is responsible for conidia wall maturation. The black square illustrates the graphic view of the wall structure of the mature conidium, including the crenulated electron-dense outer layer C1, the carbohydrate-condensed layer C3, the electron-thin layer C2, and the innermost layer C4. Note: some *Aspergillus* species lack metulae (ex. *A*. *parasiticus*), and some species can have both metulae-phialides or phialides-only conidiophores (ex. some *A*. *flavus* variants) [11]. (B) Unrooted phylogeny of WetA amino acid sequences of *A*. *flavus* NRRL3357 XP_002383329.1 (AFL), *A*. *fumigatus* Af293 XP_751508.1 (AFU), *A*. *nidulans* FGSC4 XP_659541.1 (ANI), *A*. *oryzae* RIP40 XP_001816745.1 (AOR), *Penicillium chrysogenum* Wisconsin 54–1255 XP_002564365.1 (PCH), *P*. *digitatum* Pd1 XP_014534725.1 (PDI), *Fusarium graminearum* PH-1 I1S0E2.2 (FGR), and *Beauveria bassiana* ARSEF 2860 XP_008599445.1 (BBA) [12–18]. The sequences were aligned using MAFFT, version 7.1.5 [19]. The WetA protein phylogeny was calculated using the maximum likelihood optimality criterion, as implemented in PAUP [20], version 4.0a152; we used the WAG model of amino acid evolution [21], with empirical amino acid frequencies and allowing for rate heterogeneity among sites. Values near internal branches correspond to bootstrap support values (only values above 70% are shown). Branch lengths correspond to the estimated number of amino acid substitutions per site–the internal branch leading to the FGR and BBA sequences has been truncated for optimal visualization. (C) The predicted WetA protein architecture. The red circle and the red box represent the transcription activation domain (TAD) which was predicted by 9aaTAD using the “Less stringent Pattern” setting [22]. The blue diamond and the blue box represent the nuclear localization signal (NLS) predicted by NLStradamus using the 4 state HMM static model [23]. The orange rectangle and the orange box represent the ESC1/WetA-related domain (PTHR22934) predicted by the PANTHER classification system [24]. The consensus sequence and the consensus histogram are shown under the amino acid sequence multiple sequence alignment.

The asexual reproductive cycle of *Aspergillus* fungi can be divided into two distinct phases: growth and development. The growth phase involves germination of the conidium and formation of an undifferentiated network of hyphal cells that form the mycelium. Once nutritional resources begin to be limiting, some of the hyphal cells stop mycelial growth and begin asexual development (conidiation) by forming complex structures called conidiophores that bear multiple chains of conidia (Fig 1A), completing the asexual reproductive cycle [9]. Conidiation in *Aspergillus* involves distinct morphological and chemical processes [9]. For example, a key morphological process is the formation of a large number of conidia with specialized cell walls. Similarly, key primary metabolic processes include the acquisition of pigments and massive biogenesis of trehalose within the spore (up to 15% of dry weight), providing protection and long-term viability [10].

A key and essential step for conidiophore development in *Aspergillus* is activation of *brlA*, which encodes a C_2_H_2_ zinc finger transcription factor (TF) (Fig 1A) [9,25]. Further genetic and biochemical studies identified *abaA* and *wetA* as genes that are also important for conidiation. The *abaA* gene, activated by BrlA during the middle stages of conidiation, has been reported to function in the differentiation and functionality of the cells that produce conidia, which are known as phialides [26,27]. The *wetA* gene is activated by AbaA at the late phase of conidiation and functions in the synthesis of crucial conidial wall components, such as the inner C4 layer, which makes conidia impermeable and mature [28,29]. These three genes define a central regulatory pathway that acts in concert with other genes to control conidiation-specific gene expression and determine the order of gene activation during conidiophore development and spore maturation [30].

In this report, we have characterised the functions of WetA (wet-white A) in an aflatoxigenic *A*. *flavus* strain (NRRL3357) employing genetic, analytical, and genomic approaches as a way to better understand the developmental and chemical biology of this important plant pathogen that dramatically impacts human health. Similar to what has been described in *A*. *nidulans* [29], the deletion (Δ) of *wetA* in *A*. *flavus* resulted in various defects, including the formation of wet-white conidia that take up water and autolyze rather than undergoing the final stages of maturation. *A*. *flavus* Δ*wetA* conidia are defective in the formation of a complex cell wall and lack pigments. TEM analysis indicates that many of the Δ*wetA* mutant conidia are misshaped and lack cytoplasm. Moreover, the Δ*wetA* mutant conidia lack trehalose and are highly sensitive to heat and oxidative stress. Importantly, WetA is also necessary for proper vegetative growth and AFB production. RNA-seq analyses of conidia indicate over 5,700 genes are differentially expressed between wild-type (WT) and the mutant conidia including 160 genes predicted to encode (putative) TFs, indicating a global regulatory role of WetA in conidiogenesis. Collectively, we propose that the evolutionarily conserved WetA protein plays a global regulatory role in governing growth, development, and bridging spore differentiation and survival in *A*. *flavus*.

## Materials and methods

### Strains, media, and culture conditions

*Aspergillus* strains used in this study are listed in S1 Table. The fungal strains were grown on minimal medium (MM) with appropriate supplements as described previously [31,32] and incubated at 30°C. To determine the number of conidia, WT and mutant strains were point-inoculated and grown on solid MM at 30°C for 2 days. The conidia were collected in ddH_2_O from the entire colony and counted using a hemocytometer for further experiments. For liquid submerged cultures, conidia of WT and mutant strains were inoculated in liquid MM and incubated at 30°C, 220 rpm. When comparing the conidiation levels, the conidia were collected from mycelial mats of the same size and were counted 2 days after conidiation induction. Conidiation induction was performed as previously described [33]. *Escherichia coli* strains, DH5α and BL21 (DE3), were grown in Luria-Bertani medium with ampicillin (50 mg/ml) for plasmid amplification.

### Generation of *wetA* deletion and complemented strains

The oligonucleotides used in this study are listed in S1 Table. Double-joint PCR was used to generate the deletion constructs of *wetA* [34]. Briefly, the deletion constructs containing *A*. *fumigatus pyrG* marker with 5’ and 3’ flanking regions of *wetA* were introduced into the recipient strain NRRL3357.5 [35]. To generate complemented strains, a WT *wetA* gene region including its upstream 2 kb region and downstream 1 kb region was amplified and introduced into the recipient strain. Multiple *wetA* deletion mutants (Δ*wetA*) in *A*. *flavus* were generated, which all behaved the same in every assay tested. We also generated three independent complemented strains (C’*wetA*), and they all behaved identically to one another as well. We chose TMY1 (Δ*wetA*) and TMY2 (C’*wetA*) as the testing strains for further experiments.

### Nucleic acid manipulation

To isolate genomic DNA, about 10^6^ conidia of relevant strains were inoculated in 2 ml liquid MM and stationary cultures at 30°C for 2 days. The mycelial mat was collected, squeeze-dried, and genomic DNA was isolated as described [34]. Total RNA isolation for Northern blot analyses was performed as described [33,34,36]. For RNA-seq, 2-day-old conidia of WT and Δ*wetA* strains were harvested from solid MM. Total RNA was extracted and submitted to ProteinCT Biotechnologies (Madison, WI) and the University of Wisconsin Biotechnology Center (Madison, WI) for library preparation and sequencing.

### Conidia viability, autolysis, and stress response test

To check conidial viability, 2-day-old conidia of WT, Δ*wetA*, and C’*wetA* strains were collected and spread onto solid MM and cultured at 30°C. At 2-, 5-, 7-, 14-, and 20-days post-inoculation, conidia were collected from MM plates, and approximately 200 conidia were inoculated onto solid MM and cultured until colonies appeared. Survival rate was calculated as the ratio of the actual colony number to expected colony number. To test for conidial autolysis, approximately 100 conidia of WT, Δ*wetA*, and C’*wetA* strains were inoculated onto solid MM and incubated at 30°C for 4, 7, and 18 days. Conidia from the entire plate were collected and counted. Relative conidial number was compared to the number of conidia derived from a 4-day-old plate of each strain.

Two-day-old conidia of WT, Δ*wetA*, and C’*wetA* strains were collected to examine stress tolerance. For thermal stress tolerance tests, conidia were incubated at 50°C for 0, 10, and 60 minutes, and then spread onto solid MM. For UV stress tolerance tests, conidia were spread on solid MM and exposed to varying UV intensities (0, 100, and 200 J/cm^2^). For osmotic stress tolerance tests, conidia were spread onto solid MM with different concentrations (0.0, 0.6, and 2.4 M) of KCl. Finally, for oxidative stress tolerance tests, conidia were spread onto solid MM with different concentrations (0, 2, and 4 mM) of H_2_O_2_. All plates were incubated at 30°C until colonies appeared. Colony numbers were counted and calculated as a percentage of the untreated control.

### Conidia content quantification

Two-day-old conidia of WT, Δ*wetA*, and C’*wetA* strains were collected. Trehalose and β-glucan quantification was performed as previously described [13,37].

### Transmission electron microscopy (TEM)

Two-day-old conidia of WT and Δ*wetA* were collected from MM plates. Sample preparation was performed as previous described [31]. TEM analyses were done by the UW Electron Microscope Facility.

### Vegetative growth rate and hyphal branching rate tests

Conidia of WT, Δ*wetA*, and C’*wetA* strains were point inoculated onto solid MM and cultured at 30°C. Colony diameter was measured daily until day 7 post inoculation. To measure the hyphal branching rate, conidia of WT and Δ*wetA* strains were inoculated and cultured for 18 hours at 30°C, 220 rpm in liquid MM (V18). The mycelium aggregates were transferred to solid MM and cultured for 8 hours (A8). The average peripheral growth unit (PGU) is defined as the distance between the first two branching points from the hyphal tips. At least 15 PGUs were measured for each strain.

### AFB1 quantification

Two-day-old conidia of WT, Δ*wetA*, and C’*wetA* strains were inoculated into 100 ml MM (10^3^/ml) and cultured for 5 days at 30°C, 220 rpm. Individual liquid cultures were filtered by a single layer of Miracloth. The mycelium aggregates were squeezed with a paper towel to remove as much of the medium as possible. The mycelium mat was placed in a 65°C oven for 2 hours and then its dry weight was quantified. Two milliliters of the culture (with or without filtration to remove the mycelium) were mixed with an equal volume of chloroform, vigorously vortexed, and centrifuged. The chloroform (bottom) layer (750 μl) of each sample was transferred and evaporated in a glass tube overnight. Then each dried sample was dissolved in 500 μl methanol anhydrous and filtrated by a 0.45 μm filter syringe into HPLC vials. Each sample was injected into the HPLC (Agilent 1200 series) at a flow rate of 0.8ml/min with water:acetonitrile anhydrous:methanol anhydrous (20:40:40, v/v), and AFB was detected by a diode array detector at a wavelength of 365 nm [38]. The injected volume was 10 μl and the separation was performed via Agilent HPLC column Zorbax Eclipse XDB-C18, 5um, 4.6 x 250mm cart.

### RNA sequencing

A strand-specific library was prepared from total RNA using the Illumina TruSeq Strand-specific RNA sample preparation system. Briefly, mRNA was extracted from total RNA using poly-A selection, followed by RNA fragmentation. The strand-specific library was constructed by first-strand cDNA synthesis using random primers, sample cleanup, and second-strand synthesis using DNA Polymerase I and RNase H. A single 'A' base was added to the cDNA fragments followed by ligation of the adapters. Final cDNA library was achieved by further purification and enrichment with PCR, then quality checked using a Bioanalyzer 2100. The library was sequenced (SE100bp) using the Illumina HiSeq2500, and over 19 million high-quality reads per sample were achieved. All RNA-seq data files are available from the NCBI Gene Expression Omnibus database (Accession number: GSE95711).

### Gene expression analysis

The quality of the raw sequence reads was verified using version 0.11.5 of FastQC [39]. The *A*. *flavus* genome and gene annotations were downloaded from NCBI (GCF_000006275.2_JCVI-afl1-v2.0_genomic.gff) and used for mapping. Mapping of the raw sequence reads to the genome was carried out with version 2.1.1 of Tophat2 [40], and the default options were used except for the maximum intron length was set to 4,000 bases (—max-intron-length 4000). Most (77–93%) of the reads from each of the samples mapped to the *A*. *flavus* genome. The alignment BAM files were compared against the gene annotation GFF file, and raw counts for the number of reads mapping to each gene were generated using version 0.6.1 of HTSeq-count [41]. Approximately 70–80% of mappable reads from each of the samples could be assigned to genes. Differential expression analysis of the raw counts was carried out using version 1.14.1 of DESeq2 [42]. Genes were considered differentially expressed between the WT and Δ*wetA* conidia if their adjusted p-value was less than 0.05 and their log_2_ fold change was less than -1 or greater than 1.

### Functional enrichment analysis

GO annotations for *A*. *flavus* genes were downloaded from the AmiGO 2 website (version 2.4) on February 8, 2017 [43], and terms enriched in either the WetA-activated or -repressed gene lists were detected using version 3.0.3 of the BiNGO application [44] for Cytoscape (version 3.4.0) [45]. Version 1-26-17 of the Gene Ontology (go.obo) [46] was used to establish GO term relationships. GO terms were considered enriched if their p-value, following the Benjamini-Hochberg correction as implemented in BiNGO, was less than 0.05.

## Results

### Protein sequence features of WetA in *A*. *flavus*

The *A*. *flavus wetA* (XM_002383288.1) ORF comprises 1,692 bp with no introns and is predicted to encode a 563 amino-acid (aa) long protein. BlastP analysis against eight previously characterized WetA amino acid sequences reveals that *A*. *flavus* WetA has 99%, 61%, 57%, 53%, 53%, 68%, and 35% aa identity with WetA of *A*. *oryzae*, *A*. *fumigatus*, *A*. *nidulans*, *Penicillium chrysogenum*, *P*. *digitatum*, *Beauveria bassiana*, and *Fusarium graminearum*, respectively. Phylogenetic analysis revealed that *A*. *flavus* WetA is nested within a clade that also contains the *A*. *oryzae*, *A*. *nidulans*, *P*. *digitatum*, and *P*. *chrysogenum* WetA sequences, while *B*. *bassiana* WetA clusters with the *F*. *graminearum* sequence. Moreover, *A*. *flavus* WetA is almost identical to *A*. *oryzae* WetA and is relatively more similar to *A*. *fumigatus* WetA compared to *A*. *nidulans* WetA (Fig 1B). *A*. *flavus* WetA, along with all other WetA proteins included in our analyses, has a conserved 51-aa-length ESC1/WetA-related domain (PTHR22934: SF29) with the putative DNA-binding ability originating near the C terminus (Fig 1C) [14,47]. This highly conserved domain is further predicted by 9aaTAD and NLStradamus [22,23] to contain a 9-aa-length transcription activation domain (TAD) and a 16-aa-length nuclear localization signal (NLS), suggesting that WetA is a potential TF (Fig 1C).

### The role of WetA in conidia

**Fig 2:**
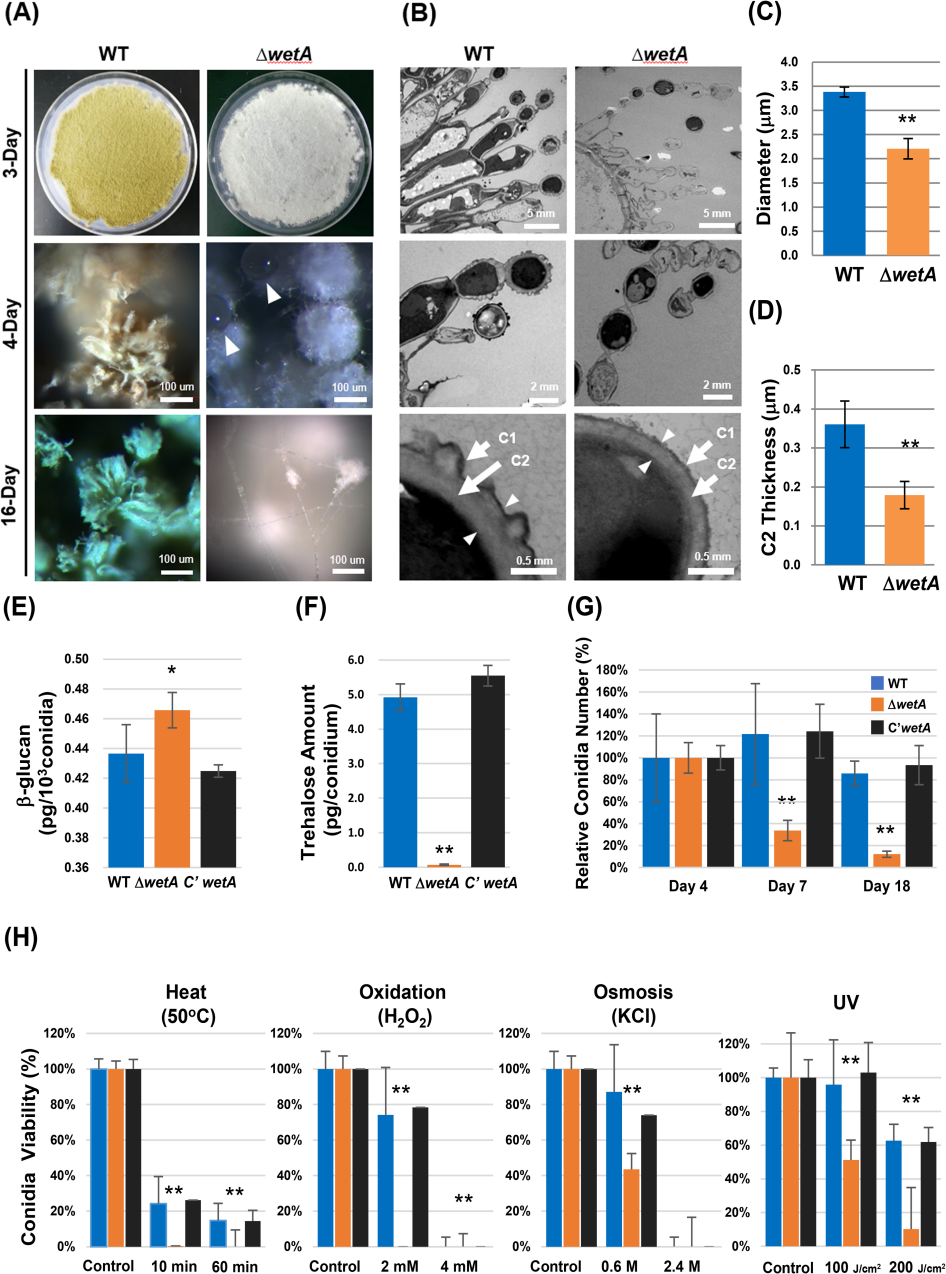
WetA is necessary for the proper formation of conidia in *Aspergillus flavus*. (A) Phenotypes of WT (NRRL3357), Δ*wetA*, and C’*wetA* grown on solid MM at 30°C for 3, 4, 16 days after asexual induction. The white triangles indicate the liquid droplets formed on the autolyzing conidiophores of Δ*wetA* strain. (B) TEM images of 2-day-old conidiophores/conidia of WT and Δ*wetA* strains. Note: the remnant of lysed conidia formed a wet-vesicle-like structure on the top of the conidia chain, and most of the conidiophore/conidia contents were lost in the Δ*wetA* strain. The bottom panels show the conidia wall structures of WT and Δ*wetA* strains. Arrows indicate the locations of the C1 and C2 layers while the arrowheads indicate the C2 layer thickness. (C, D) The average diameter of conidia and thickness of the C2 layer of WT and Δ*wetA* conidia. At least seven WT and Δ*wetA* intact conidia from different sample slices were measured. (E, F) Quantification of conidia content (β-(1,3)-glucan (E) and trehalose (F)) of WT, Δ*wetA*, and C’*wetA* 2-day-old conidia The error bars indicate one standard deviation from the mean and the asterisks the level of significance (*, *p* < 0.05; **, *p* < 0.01). (G) The relative viability of WT, Δ*wetA*, and C’*wetA* conidia grown on solid MM at 30°C for 4, 7, 18 days after inoculation. The conidial viability at day 4 of each strain was set as 100%. ** (*p* < 0.01). The error bars indicate one standard deviation from the mean viability of triplicates. (H) Tolerance of WT, Δ*wetA*, and C’*wetA* 2-day-old conidia to heat (50°C), oxidative (H_2_O_2_), osmotic (KCl), and UV stresses. The control indicates untreated conidia. The viability of the untreated conidia of each strain was set as 100%. ** (*p* < 0.01). The error bars indicate one standard deviation from the mean viability of triplicates.

To understand the biological functions of WetA, we generated multiple *wetA* deletion mutants and complement strains in *A*. *flavus*. The *wetA* null mutant forms colourless (white) conidia which start to autolyze (wet) and collapse at 2 to 3 days after conidiation, forming an aggregated sphere structure (Fig 2A).

To check the detailed structural defects of the Δ*wetA* conidia, we carried out transmission electron microscopy (TEM) of conidiophores of WT and Δ*wetA* strains. As shown in Fig 2B, while WT formed intact conidial chains, the Δ*wetA* mutant showed fewer intact conidia and a high number of lysed conidial remnants. The WT conidial diameter is about 153% longer than that observed with Δ*wetA* (Fig 2C). Moreover, the WT conidium shows a crenulated electron-opaque outer layer (C1) and an electron-translucent inner layer (C2), as reported in *A*. *nidulans* and *A*. *fumigatus* [13,29]. Although Δ*wetA* conidium forms the C1 and C2 layers, the C1 outer layer is smooth and the C2 layer is more condensed and thinner than that of WT (Fig 2D).

Although the TEM results show that the intact Δ*wetA* conidium is as electron-opaque as the WT conidium, the intact Δ*wetA* conidium contains more β-(1,3)-glucan but less trehalose when compared to the WT conidium (Fig 2E and 2F).

Somewhat surprisingly, while the loss of *wetA* leads to systemic defects in conidia, about 10% of the total Δ*wetA* conidia appears to be intact even at day 16 (Fig 2A). Besides, the survivors exert consistent phenotypes with the original Δ*wetA* mutants. However, as the majority of the Δ*wetA* conidia autolyze and disintegrate, the total number of the Δ*wetA* conidia is dramatically decreased at day 7 and 18 post inoculation, whereas no significant changes in the viability of WT and C’*wetA* conidia were observable even at day 18 (Fig 2A and 2G). Finally, we examined whether the Δ*wetA* conidia show altered responses to various stresses. As shown in Fig 2H, the *wetA* null conidia are sensitive to osmotic (KCl) and oxidative (H_2_O_2_) stresses, and highly sensitive to heat (50°C) and UV stress (Fig 2H). Taken together, these results suggest that WetA plays an essential role in the proper maturation and stress tolerance of conidia in *A*. *flavus*.

### The roles of WetA in growth, hyphal branching, AFB production, and developmental control

**Fig 3:**
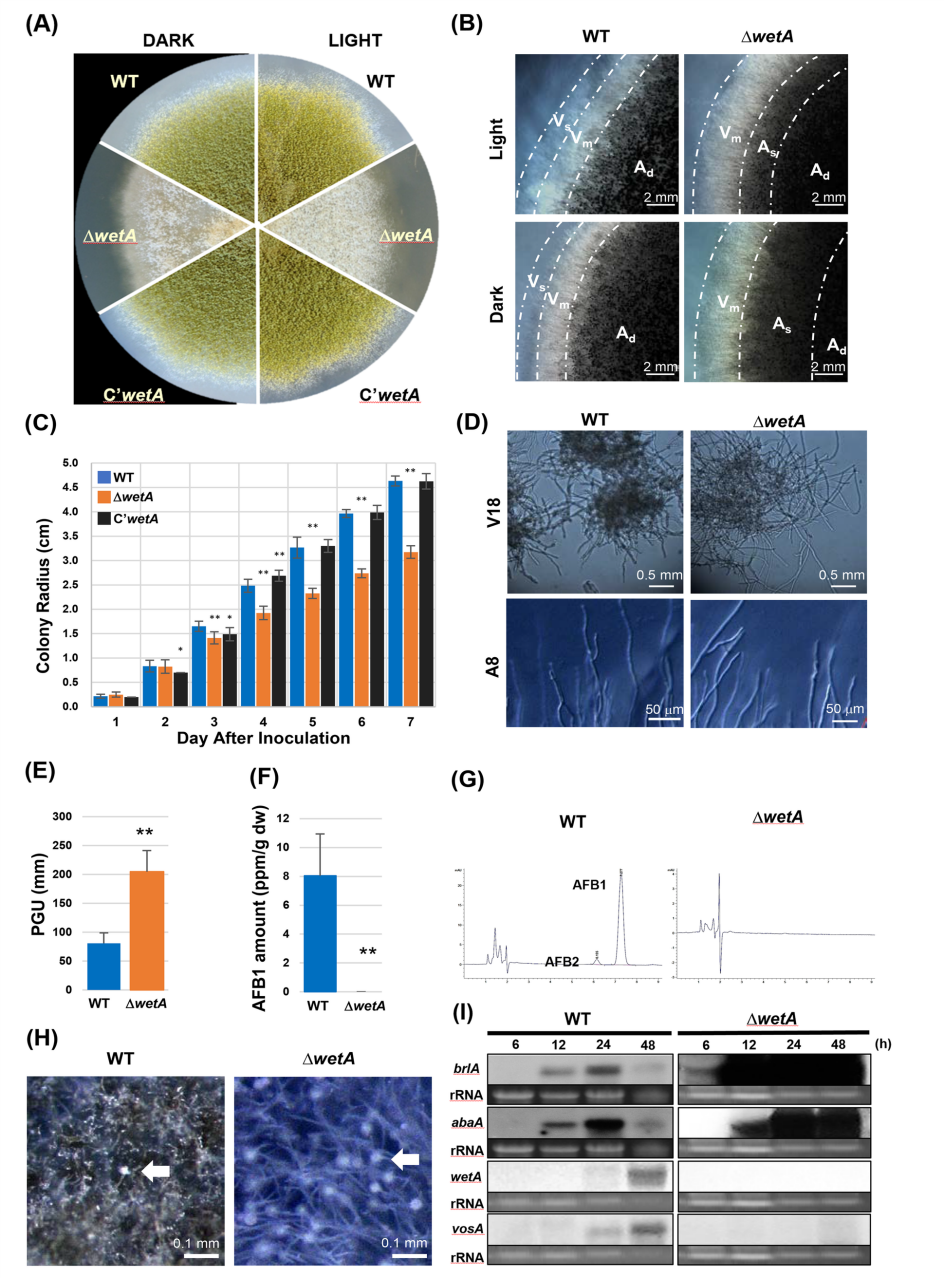
Multiple roles of WetA. (A-C) WetA affects vegetative growth. (A) The colony image of WT, Δ*wetA*, and C’*wetA* strains on solid MM at 5 days after point inoculation under light and dark conditions. (B) Colony edge image of WT and Δ*wetA* strains under light and dark conditions. V_s_: single-layer vegetative hyphae region. V_m_: multi-layer vegetative region. A_s_: sparse aerial hyphae region. A_d_: dense aerial hyphae region. (C) Colony growth rates of WT, Δ*wetA*, and C’*wetA* strains after point inoculation on solid MM. The error bars indicate one standard deviation. * (*p* < 0.05) and ** (*p* < 0.01). (D, E) Hyphal branching rates of WT and Δ*wetA* strains. (D) Microscopy images show WetA regulates hyphal branching. Loss of *wetA* leads to reduced hyphal branching rate in both solid and submerged cultures. (E) Average PGU values of A8. ** (*p* < 0.01). The error bars indicate one standard deviation. (F, G) Aflatoxin quantification by HPLC of WT and Δ*wetA* submerged culture after 5-days cultivation. (F) AFB1 amount (per g dry weight) in WT and Δ*wetA* vegetative cells. ** (*p* < 0.01). The error bars indicate one standard deviation. (G) The HPLC chromatograms of AFB1 and AFB2 in the culture medium of WT and Δ*wetA* strains. (H) WT and Δ*wetA* strains were induced for asexual development and observed after 8 h incubation at 30°C on solid MM plate. The white arrows indicate conidiophores. Note: the abundant conidiophore formation in Δ*wetA* culture. (I) Northern blot analysis of *brlA*, *abaA*, *wetA*, and *vosA* mRNA levels in WT and Δ*wetA* strains at 6, 12, 24, 48 h after conidiation induction.

We further tested the roles of WetA in governing other biological processes. We found that, in addition to conidiation, WetA is associated with proper hyphal growth. The WT and C’*wetA* strains showed higher colony growth rate than the Δ*wetA* mutant on solid minimal medium (MM), regardless of the presence or absence of the light (Fig 3A and 3C). However, WetA appears to affect conidiation and hyphal development in response to light. Under dark condition, the Δ*wetA* colony exhibits highly reduced conidiation levels (Fig 3A). The WT colony edge can be divided into three regions, the single layer vegetative hyphae region (V_s_), the multi-layer vegetative hyphae region (V_m_), and the dense aerial hyphae region (A_d_). In comparison to WT, the Δ*wetA* colony’s edge does not contain the V_s_ region and instead has a sparse aerial hyphae region (A_s_) between the V_m_ and A_d_ regions (Fig 3B). In addition, the A_s_ region of the Δ*wetA* colony is expanded when grown in the dark environment (Fig 3A and 3B and S1 Fig). Furthermore, the absence of *wetA* results in about 2.5-fold lower hyphal branching rate in both the submerged culture and the solid culture (Fig 3D and 3E), which is consistent with our previous observation of the same phenotype in Δ*wetA* in *A*. *fumigatus* [13]. We noticed that both the apical branching and lateral branching rates were reduced in Δ*wetA* mutants, suggesting that WetA is involved in both branching systems. To further elucidate the role of WetA, we examined AFB production in WT, Δ*wetA*, and C’*wetA* strains shake-cultured for 5 days by HPLC. The results show that the Δ*wetA* strain was unable to produce AFB1 and AFB2 in the submerged culture (Fig 3F and 3G).

We found that the absence of *wetA* resulted in precocious conidiophore development. The Δ*wetA* mutant started to generate abundant conidiophores at 6 h post asexual developmental induction, an hour earlier than WT and complement strains, and by 8 hours the difference in conidiophore production was striking (Fig 3H). This observation was corroborated by Northern blot analysis. As shown in Fig 3I, loss of *wetA* leads to early accumulation of mRNA from the asexual reproduction-inducing gene *brlA* at 6 h after asexual developmental induction, whereas *brlA* transcript only started to accumulate at 12 h after induction in WT. In WT, mRNA levels of *brlA* and *abaA*, another conidia-inducing regulator, reach their highest at 24 h and drop at 48 h after induction. In contrast, transcript levels of *brlA* and *abaA* dramatically increase at 12 h and stay at high levels at 24 and 48 h after asexual induction in the Δ*wetA* mutant. In WT, the mRNA for the regulator *vosA* started to accumulate at 24 h post-induction, while *vosA* mRNA accumulation was hardly detected in Δ*wetA* strain (Fig 3I). These results indicate that in *A*. *flavus*, WetA is a key feedback negative controller of *brlA* expression and conidiation. Overall, our data suggest that WetA plays multiple roles in cellular and chemical development in *A*. *flavus*.

### Genome-wide expression analyses in conidia

**Fig 4:**
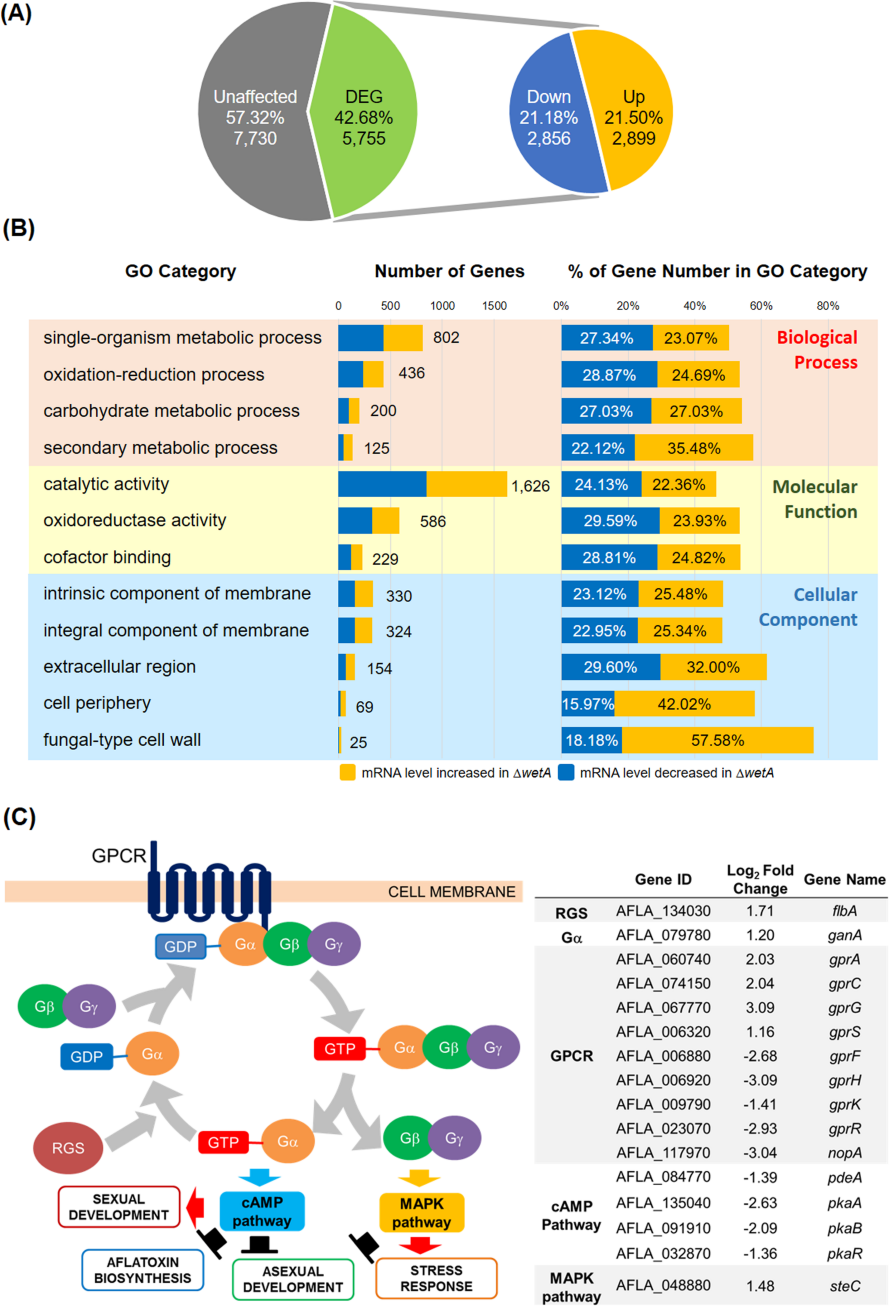
RNA-seq analyses of conidia. (A) The numbers of genes whose mRNA levels were similar (Unaffected, grey), or different between WT and Δ*wetA* conidia (DEG, green), with down (blue) and up (yellow) in the Δ*wetA* conidia compared to WT. A DEG is defined by having a > 2-fold change of mRNA levels between WT and Δ*wetA* conidia and an adjusted *p*-value of less than 0.05. (B) Functional categories of DEGs in conidia. The yellow bars represent genes whose mRNA levels increased in the Δ*wetA* conidia, whereas the blue bars represent those genes whose mRNA levels decreased in the Δ*wetA* conidia. The pink shaded box represents the biological process GO categories; the yellow shaded box represents the molecular function GO categories; the blue shaded box represents the cellular component GO categories. “Number of Genes”: the total number of DEGs assigned to the specific GO category. “% of Gene Number in GO”: the number of DEGs divided by the total number of genes assigned to the specific GO category in the genome as a whole. (C) The schematic diagram and mRNA expression profile of the G-protein regulatory pathways controlling development, stress response, and aflatoxin biosynthesis.

To shed more light on the multiple regulatory roles WetA appeared to play in *A*. *flavus* biology, we carried out genome-wide expression analyses in WT and mutant conidia using RNA-seq. Poly-A mRNA from three technical replicates of 2-day-old conidia of WT and Δ*wetA* strains were purified and sequenced as described in the methods section; one technical replicate of the Δ*wetA* was discarded following failure in multiple quality control analyses. Examination of global gene expression differences between the WT and mutant *wetA* indicate that WetA plays a broad regulatory role in conidia. Out of the 13,485 mapped *A*. *flavus* genes, 5,755 (42.68% of the total) showed differential accumulation of mRNAs in the Δ*wetA* conidia in comparison to WT conidia. Among 5,755 differentially expressed genes (DEGs), mRNA levels of 2,856 (21.18%) genes were lower (Down) in the Δ*wetA* conidia compared to WT conidia, and those of 2,899 (21.50%) genes were higher (Up) in the Δ*wetA* conidia compared to WT conidia (Fig 4A).

Functional category analysis was carried out by determining Gene Ontology (GO) terms that were enriched in DEGs. The top enriched biological process GO categories are “single-organism metabolic process”, “oxidation-reduction process”, “carbohydrate metabolic process”, and “secondary metabolic process”. The top enriched molecular function GO categories are “catalytic activity”, “oxidoreductase activity”, and “cofactor binding”. The top enriched cellular component GO categories are “intrinsic component of membrane”, “integral component of membrane”, “extracellular region”, “cell periphery”, and “fungal-type cell wall” (Fig 4B). Of note, over 50% of all genes in the *A*. *flavus* genome annotated with the GO terms “carbohydrate metabolic process” (54.05%), “secondary metabolic process” (57.60%), and “fungal-type cell wall” (75.76%), were regulated by WetA in our RNA-seq data, consistent with our phenotypic data (Fig 4B). The top enriched GO categories for genes whose mRNA levels decreased or increased in the Δ*wetA* conidia are listed in S2 and S3 Tables, and the top 100 DEGs with decreased/increased mRNA accumulation levels in the Δ*wetA* conidia are listed in S4 and S5 Tables, respectively.

To explore the molecular roles of WetA in conidiation, we checked mRNA levels of those genes assigned to the GO term “Asexual Development” (GO:0019954) and other known genes related to asexual development [16]. In total, 77 genes related to asexual development are differentially expressed by the absence of WetA: 29 genes (37.66%) and 48 genes (62.34%) showed decreased and increased mRNA levels in the Δ*wetA* conidia, respectively (see Table 1 and S6 Table). These data corroborate our working hypothesis that WetA is an important feedback regulator of conidiation, likely by activating several conidiation repressors and repressing key conidiation activators.

**Table 1:** DEGs of interest.

	mRNA level decreased in Δ*wetA*	mRNA level increased in Δ*wetA*
**Asexual Development**	*argB*, *bem1*, *dewA*, *fluG*, *fphA*, *kex1*, *nce102*, *nudG*, *osaA*, *pbcR*, *pkaA*, *pkaB*, *pkaR*, *ppoA*, *ppoB*, *rft1*, *rhbA*, *ricA*, *rodB*, *sfgA*, *swoM*, *tcpA*, *tmpA*, *tpsA ortholog*, *tpsC*, *veA*, *vosA*, *wA/pksP*, *wetA*	*abaA*, *ams1*, *atg1*, *atgH*, *brlA*, *cch1*, *chsA*, *chsB*, *chsE*, *chsF*, *chsG*, *crzA*, *esdC*, *fbx15*, *figA*, *flbA*, *flbC*, *ime2*, *llmB*, *llmF*, *medA*, *midA*, *mob1*, *msdS*, *mtfA*, *nsdC*, *nsdD*, *nudA*, *odeA*, *pac2/osaB*, *pcl1*, *phnA*, *ppoC*, *ppoD*, *prpA*, *rgdA*, *rho1*, *rodA*, *sidB*, *sltA*, *ssc1*, *steC*, *stuA*, *ugtA*, *vapA*, *wsc1*, *wsc3*, *zipA*
Transcription Factor*	*aflR*, *aflYd*, *amdA*, *amdR*, *aro80*, *atf21*, *clrA*, *ctf1B*, *fcr1*, *fkh1*, *galX*, *metR*, *nosA*, *nscR*, *pbcR*, *pcaG*, *prnA*, *rdr1*, *regA*, *scfA*, *sdrA*, *sfgA*, *sfp1*, *silA*	*abaA*, *amdX*, *aoiH*, *brlA*, *cnjB*, *cpcA*, *crzA*, *devR/hpa3*, *egd1*, *egr2*, *flbC*, *glcD*, *hacA*, *mtfA*, *ndtA*, *nsdC*, *nsdD*, *pacC*, *rap1*, *rfeB*, *rfeG*, *rgdA*, *rpn4*, *seb1*, *sltA*, *srbA*, *steA*, *stuA*, *zipA*
**Aflatoxin Cluster**	*aflA*, *aflR*, *aflS*, *aflYd*	*aflB*, *aflC*, *aflT*, *aflU*, *aflW*, *alfYa*

^*^only those genes with annotation are listed

As the GO terms “intrinsic component of membrane” and “integral component of membrane” were enriched in functional analysis, we further examined mRNA levels of the membrane-receptor-encoded-genes like G-protein coupled receptors (GPCRs). A heterotrimeric G-protein pathway can be involved in the activation of cAMP pathways and MAPK pathways, which then lead to the repression of asexual development [48]. The *A*. *flavus* genome contains 15 GPCRs [49], of which 9 showed altered mRNA levels (4 up, 5 down) in the Δ*wetA* conidia compared to WT (Fig 4C). The *ganA* gene predicted to encode a G protein alpha subunit (Gα), and the *flbA* gene predicted to encode a regulator of G protein signaling (RGS) protein showed increased mRNA levels in the Δ*wetA* conidia (Fig 4C). Genes involved in the cAMP pathway like *pdeA*, *pkaA*, *pkaB*, and *pkaR*, showed decreased mRNA levels in the Δ*wetA* conidia (Fig 4C). Gene involved in the MAPK pathway, *steC*, show increased mRNA levels in the Δ*wetA* conidia (Fig 4C). Our results suggest that the WetA-mediated regulation may be associated with signal transduction pathways.

**Fig 5:**
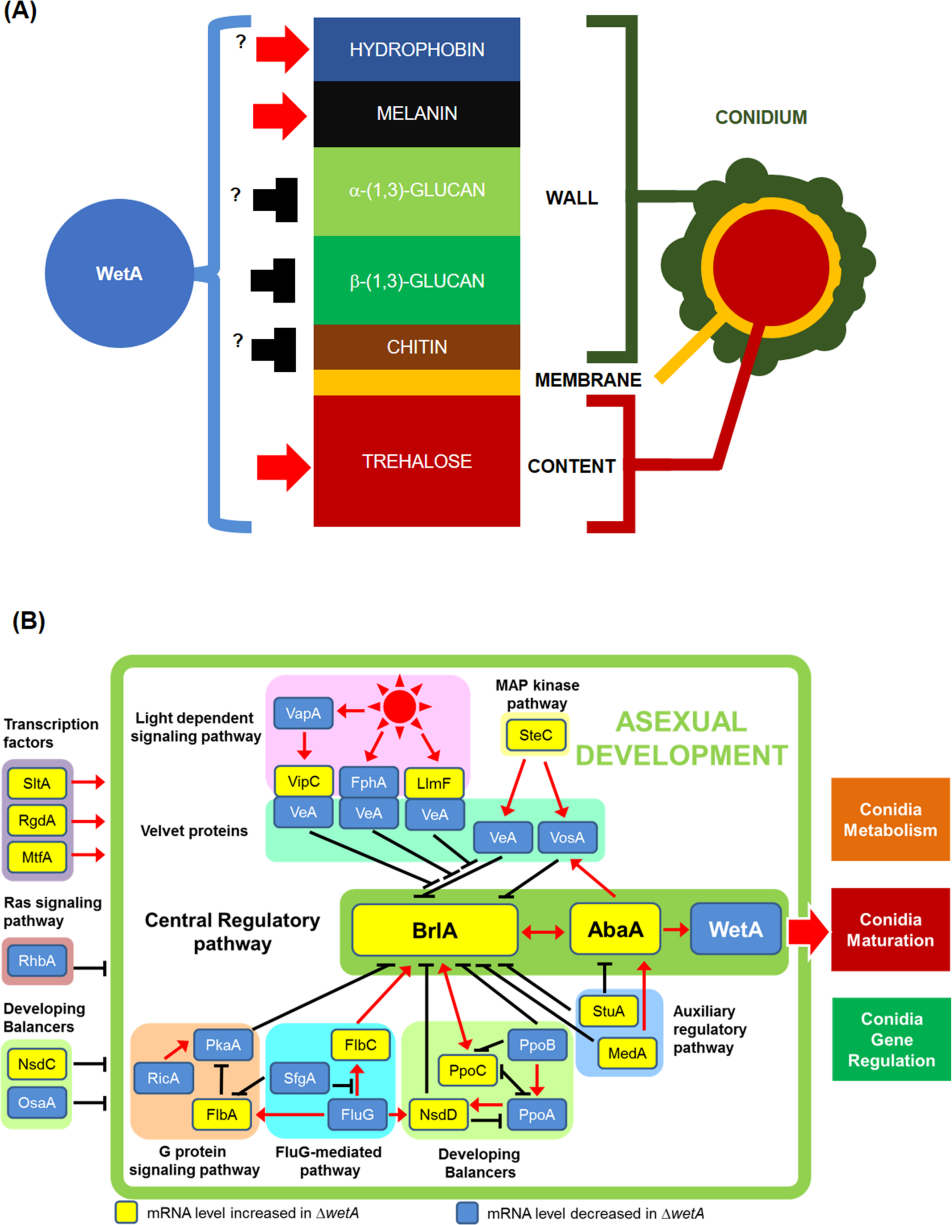
Summary of WetA functions and a model for WetA-mediated developmental regulation in *A*. *flavus*. (A) Schematic diagram of the WetA-mediated regulatory model of conidia architecture. The question mark indicates that the WetA-mediated activation/repression function needs to be verified by further experiments. (B) A comprehensive model for WetA-mediated regulation of asexual development based on transcriptomic, genetic, and biochemical data. In this model, those genes with increased and decreased mRNA levels in the Δ*wetA* conidia are labeled in yellow (WetA-inhibited) and blue (WetA-activated), respectively. The functions and references of each gene in this model are listed in S9 Table.

Next, we checked the expression levels of genes involved in conidia content and conidial wall integrity. As shown in Table 2, genes associated with the biosynthesis of trehalose, melanin, and hydrophobins, as well as the degradation of β-glucan, showed decreased mRNA levels in the Δ*wetA* conidia. Conversely, mRNA levels of genes associated with biosynthesis of chitin and β-(1,3)-glucan were increased in the Δ*wetA* conidia. These transcriptomic data and our direct measurement of β-glucan and trehalose indicate that WetA governs the integrity of conidia by coordinating intracellular contents and conidial wall biogenesis (Fig 5A, Table 2 and S7 Table).

**Table 2:** DEGs involved in spore maturation.

	mRNA level decreased in Δ*wetA*	mRNA level increased in Δ*wetA*
**Trehalose Biosynthesis**	*tppB*, *tppC*, *rfaB*, *ccg-9*, *tpsC*, AFLA_087630	
**Trehalose Degradation**	*treA*	
**Chitin Biosynthesis**		*chsA*, *chsC*, *chsE*, *chsF*, *chsG*, *chsZ*
**Chitin Degradation**		*chiA*, *chiB*, *cts2*, *ctcB*, *nagA*, AFLA_031380, AFLA_107830, AFLA_057680
**α-(1,3)-glucan Biosynthesis**	*ags1*	*ags2*
**α-(1,3)-glucan Degradation**	*agnD*, *agnE*	AFLA_091790
**β-(1,3)-glucan Biosynthesis**		*fksP*, *gel1*, *gel2*, *gel4*, *gel5*, *gel6*, *gel7*, AFLA_107790, AFLA_064920
**β-(1,3)-glucan Degradation**	*bgt1*, *exg1*, AFLA_023650	*engl1*, *eng3*, *eng4*, *eng8*, *exg0*, *exg2*
**Melanin Biosynthesis**	*wA (pksP)*, *ayg1*	
**Hydrophobin**	*dewA*, *rodB*, AFLA_063080, AFLA_098980	*rodA*, AFLA_094600

The GO term “secondary metabolic process” is enriched in the WetA-influenced transcriptome. We examined the differentially expressed genes belonging to secondary metabolite gene clusters (SMG clusters). We predicted the *A*. *flavus* SMG clusters with antiSMASH and used the cluster boundaries identified by Inglis *et al*. for *A*. *oryzae* clusters if they were conserved in *A*. *flavus* [50,51]. If the clusters were not identified by Inglis *et al*., we used the default boundaries provided by antiSMASH. There are 660 SMGs distributed in the 74 SMG clusters in *A*. *flavus* (S8 Table). We found that 306 genes (46.37%) distributed in 68 SMG clusters (92%) showed altered mRNA levels in the Δ*wetA* conidia (136 down and 170 up, see S8 Table). All of the genes located in Clusters 23, 35, 41, 46, 48, 52, 54, and 71 showed altered mRNA levels in the Δ*wetA* conidia. Interestingly, all genes in Clusters 23 and 52 showed decreased mRNA levels in the Δ*wetA* conidia, whereas all genes in Cluster 71 showed increased mRNA levels the Δ*wetA* conidia. These data indicate an important role of WetA governing secondary metabolic chemical development in conidia.

Finally, we focused on putative TFs showing altered mRNA levels in the Δ*wetA* conidia as this functional category was enriched among WetA-induced DEGs (S2 Table. We found that 160 genes predicted to encode TFs exhibited altered mRNA accumulation in the presence and absence of WetA in conidia: 100 (62.50%) showed decreased mRNA levels and 60 (37.50%) showed increased mRNA levels in the Δ*wetA* conidia. Approximately 80% of these putative TFs have a zinc binding domain, including 18 TFs with a C_2_H_2_ domain and 64 TFs with a Zn(II)_2_Cys_6_ (or C_6_) domain (Table 1 and S10 Table); important classes for the regulation of fungal development and metabolism. Taken together, WetA governs proper expression of various signaling, regulatory, structural, and metabolic elements that coordinate cellular and chemical development of conidia.

## Discussion

Asexual development has been studied in *Aspergilli* and other fungi for many years [13–18,28–30,52–59]. In addition to *Aspergillus* fungi, the function of WetA is highly conserved in other Ascomycetes. In *P*. *digitatum*, the lack of *wetA* also results in abnormal conidia, delayed germination, and reduced stress tolerance [15]. Moreover, *P*. *chrysogenum wetA* can fully complement the *A*. *nidulans wetA* deletion mutation, suggesting that the WetA-mediated sporulation regulatory mechanisms are conserved in *A*. *nidulans* and *P*. *chrysogenum* [18]. In *F*. *graminearum*, loss of *wetA* causes deficient conidia, reduced oxidative and heat stress tolerance, and reduced chronological spore viability. *F*. *graminearum* WetA suppresses microcycle conidiation and then further maintains conidial dormancy [14]. In *B*. *bassiana*, *wetA* null mutants produce deficient conidia which are sensitive to environment stresses [17].

Previous studies suggest that WetA is responsible for activating a set of genes whose products comprise or direct the assembly of the conidial wall layers and ensure proper cytoplasmic status [28]. In *A*. *nidulans*, WetA together with AbaA activate genes which are expected to encode spore-specific functions (Class B Genes). WetA together with both BrlA and AbaA activate Class C and Class D genes, which are expected to encode phialide-specific functions [28,30,60]. However, WetA alone is sufficient to activate Class B and some Class D genes [28]. At least one gene (*wA*), whose mRNA accumulates in phialide cells instead of in conidia, is activated by WetA, indicating that WetA may regulate genes in these cells as well as in conidia [28]. Additionally, accumulation of *wetA* mRNA requires *wetA*^*+*^ activity during conidiation, suggesting that *wetA* is autogenously regulated [12,28].

WetA is functionally conserved and required in *A*. *flavus* for many aspects of its biology, including spore viability, wall integrity, and stress tolerance. Although the *wetA* null mutant forms the C2 spore wall layer as found in WT, the Δ*wetA* C2 layer is hyper-condensed in *A*. *flavus* while it fails to condense in *A*. *nidulans*, *A*. *fumigatus*, and *P*. *digitatum* [13,15] (Fig 2B and 2D). The condensation of the C2 layer along with the formation of the C3 and C4 layers are the final stage of conidial maturation, which contributes to the impermeability of the conidial wall [29].

WetA is essential for establishing the heat stress tolerance in Ascomycetes [13,15,17]. However, loss of *wetA* in different species results in variable degrees of tolerance to oxidative stress and osmotic stress. The *A*. *fumigatus* [13], *A*. *flavus* (Fig 2H), and *F*. *graminearum* [14] *wetA* deletion mutants are highly sensitive to H_2_O_2_. However, the *B*. *bassiana* [17] *wetA* deletion mutants showed WT level tolerance to H_2_O_2_ and the *P*. *digitatum* [15] *wetA* deletion mutants even showed enhanced tolerance to H_2_O_2_. Loss of *wetA* causes reduced osmotic stress tolerance in *A*. *fumigatus* [13], *P*. *digitatum* [15], and *B*. *bassiana* [17], but has not in *F*. *graminearum* [14]. Somewhat surprisingly, even though the Δ*wetA* conidial structure and stress tolerance were impaired, the small numbers of intact Δ*wetA* conidia that could be isolated showed consistant phenotypes as the WT conidia. However, only about 30% of the total Δ*wetA* conidia remain intact on day 7 after inoculation, while almost 100% of WT conidia remain intact (Fig 2G).

WetA appears to be involved in both the trehalose biosynthetic and degradation pathways in *A*. *flavus* (Fig 2F, Table 2 and S7 Table), both of which are required for conidial stress tolerance and viability. The *velvet* regulator VosA is known as the regulator which couples sporogenesis and trehalose biogenesis, and it activates *wetA* in *A*. *nidulans* [31]. Our data show that WetA also activates *vosA* in *A*. *flavus* (Table 1 and S6 Table), suggesting an inter-dependent activation between the two regulators in conidia. While the loss of *wetA* does not completely block *vosA* expression in conidia (S6 Table) and vice versa [31], almost no trehalose can be detected in the Δ*wetA* (Fig 2F) and Δ*vosA* [31] conidia, suggesting that both WetA and VosA are required for proper trehalose biosynthesis in conidia. Although WetA is required for trehalose biosynthesis in *A*. *flavus* (Fig 2F), *A*. *fumigatus* [13], and *B*. *bassiana* [17], loss of *wetA* did not alter the trehalose amount in *F*. *graminearum* [14] and *P*. *digitatum* [15].

Based on microscopy images, trehalose quantifications, and β-(1,3)-glucan quantifications from our group as well as others, studies proposed that WetA regulates conidial wall integrity and trehalose content [13–15,17,29,58]. Our RNA-seq analyses further expands our understanding of how WetA affects overall conidial wall integrity. More than 75% of the genes assigned to the Cellular Component GO category “fungal-type cell wall” in the *A*. *flavus* genome were differentially expressed in the Δ*wetA* conidia (Fig 4B), resulting in this category being statistically enriched amongst WetA-regulated genes. This suggests that WetA plays a global regulatory role in spore wall integrity. The *Aspergillus* conidial wall is composed of chitin, β-(1,3)-glucan, α-(1,3)-glucan, melanin, and hydrophobin [61] (Fig 5A). Our transcriptome analyses support the hypothesis that genes involved in the metabolic pathways of the conidial wall components are differentially expressed in the Δ*wetA* conidia (Table 2 and S7 Table). Taken together, we present a summary of the role of WetA in governing conidial maturation by regulating the metabolic pathways of trehalose and conidial wall components in *A*. *flavus* (Fig 5A). However, the WetA-mediated regulatory circuits governing maturation and stress responses of conidia among various fungal species may be genetically re-wired in each fungal species, as is the case for other proteins involved in the regulation of asexual development and secondary metabolism [62].

Our data suggest that WetA is necessary to turn off the conidiation initiation process after the formation of conidiophores. Loss of *wetA* resulted in greatly enhanced levels of *brlA* and *abaA* in *A*. *flavus* (Fig 1D, Table 1 and S6 Table) *A*. *fumigatus* [13], *P*. *digitatum* [15], and *B*. *bassiana* [17]. Moreover, our transcriptome analyses indicate that loss of *wetA* resulted in altered mRNA levels of various regulators of conidiation (Table 1 and S6 Table), implying an upstream regulatory role of WetA in conidia (Fig 5B). Consistent with this, we observed earlier conidiation in the Δ*wetA* mutant compared to WT in *A*. *flavus* (Fig 3H). However, the Δ*wetA* mutant showed delayed conidiation in *A*. *fumigatus* [13], suggesting that WetA-mediated feedback regulation of conidiation has undergone genetic rewiring in *Aspergilli*.

About 46% of genes positioned in the predicted SMG clusters showed altered mRNA levels in the Δ*wetA* conidia, including 15 genes predicted to encode polyketide synthases (PKSs) and PKS-like proteins, and 19 genes predicted to encode non-ribosomal polyketide synthases (NRPSs) and NRPS-like proteins (Table 3 and S8 Table), suggesting that WetA affects biosynthesis of several secondary metabolites in conidia. The backbone gene, *aflC*, and the transcription factor, *aflR*, of the AF cluster were differentially expressed in the Δ*wetA* conidia (Table 1 and S8 Table). Although there is no significant difference in AFB amount between WT and Δ*wetA* conidia (S2 Fig), the loss of *wetA* reduces the amount of AFB1 and AFB2 in submerged culture (Fig 3F and 3G), suggesting that WetA exerts temporal and spatial regulation of aflatoxin metabolism.

**Table 3:** DEGs within the secondary metabolite biosynthesis clusters.

**BACKBONE GENES**				
	**mRNA level decreased in Δ*wetA***		**mRNA level increased in Δ*wetA***	
	**CLUSTER**	**GENE ID**	**CLUSTER**	**GENE ID**
**PKS/PKS-LIKE**	3	AFLA_127090	6	AFLA_053870
	12	AFLA_079360	10	AFLA_010000
	20	AFLA_116220	24	AFLA_118940
	21	AFLA_116890	24	AFLA_118960
	55	AFLA_006170	50	AFLA_002900
			58	AFLA_137870
			59	AFLA_139410
			71	AFLA_060020
			71	AFLA_060010
			74	AFLA_062820
**NRPS/NRPS-LIKE**	10	AFLA_010020	11	AFLA_010620
	10	AFLA_010010	26	AFLA_119820
	23	AFLA_118440	27	AFLA_121520
	25	AFLA_119110	41	AFLA_101700
	35	AFLA_038600	44	AFLA_064240
	45	AFLA_064560	46	AFLA_066720
	52	AFLA_004450	48	AFLA_069330
	54	AFLA_005440	61	AFLA_023020
	63	AFLA_028720	69	AFLA_109430
	65	AFLA_105190		
**TRANSCRIPTION FACTORS**				
	**mRNA level decreased in Δ*wetA***		**mRNA level increased in Δ*wetA***	
	**CLUSTER**	**GENE ID**	**CLUSTER**	**GENE ID**
	18	AFLA_087810	31	AFLA_096370
	31	AFLA_096330	71	AFLA_059960
	40	AFLA_100300		
	59	AFLA_139360		
	63	AFLA_028760		
	66	AFLA_105530		

Loss of *wetA* results in reduced radial growth and lowered hyphal branching rates (Fig 3A, 3C, 3D and 3E). RNA-seq results showed about 50% of the genes in the GO categories, “hyphal tip” and “site of polarised growth”, exhibited increased mRNA levels in Δ*wetA* conidia (S3 Table). Put together, WetA may play a regulatory role in hyphal development. It is possible that WetA regulates both hyphal and conidial cell wall assembly and therefore affects both the radial growth rate and branching rate of *A*. *flavus*.

We observed differential conidial and hyphal development between light and dark conditions in the Δ*wetA* mutant (Fig 3A and 3B). Transcriptome analyses revealed that several light-sensor-encoding genes were differentially expressed in the Δ*wetA* conidia, including *fphA*, *nopA*, *gprF*, and *gprR*. FphA is a phytochrome that represses sexual development in *A*. *nidulans* under red-light induction [63,64]. NopA is a fungal opsin type GPCR that represses conidiation in *Neurospora crassa* [65,66], but its function in *Aspergilli* is still vague [49,67]. GprF and GprR repress conidiation under the dark condition [49]. Taken together, WetA may be involved in light-dependent regulatory pathways that affect conidiation and hyphal development.

Our data show that WetA plays multiple roles in governing development (Figs 2 and 3). Transcriptome analyses showed that WetA affects mRNA levels of 160 genes predicted to encode TFs and 9 genes predicted to encode GPCRs (Fig 4C, Table 1 and S10 Table). G-protein signaling governs normal growth, development, and mycotoxin production in filamentous fungi [68]. GPCRs are known to be involved in multiple cell processes, including carbon and nitrogen sensing, aflatoxin repression, germination, quorum sensing, oxylipin sensing, light sensing, and osmotic, acidic pH, ROS, and cell wall stress responses [49]. Taken together, our data suggest that the absence of WetA function results in disturbed expression of TFs and GPCRs leading to downstream pleiotropic effects. As annotation of the *A*. *flavus* genome improves to the levels of that of *A*. *nidulans*, *A*. *oryzae*, *A*. *fumigatus*, and *A*. *niger* genomes, we may identify additional putative regulators influenced by WetA in conidia.

Transcripts of *wetA* were not detectable during vegetative growth in *A*. *flavus*, as observed in *A*. *nidulans* and *A*. *fumigatus*. However, loss of *wetA* caused reduced growth rate, reduced hyphal branching rate, and reduced AFB1 production during vegetative growth (Fig 3). One possible explanation is that the WetA protein is stable and present throughout the vegetative growth phase. This was the case of *vosA*, where mRNA of *vosA* is not detectable in vegetative cells, but the VosA protein is present at a high level until conidiation occurs and is still able to repress *brlA* expression in vegetative cells [31,69]. Another explanation is that WetA regulates several signaling regulatory pathways in conidia, which keep functioning in the regulation of other biological processes during development.

In conclusion, we present a genetic model depicting the molecular mechanisms of WetA-mediated regulation in cellular and chemical development in *A*. *flavus* (Fig 5B). WetA affects the pathways of conidial content and conidial wall component metabolism, and further affects conidia viability and stress tolerance. Furthermore, WetA exerts feedback control of conidiation initiation by regulating upstream regulators of asexual development.

## Supporting information

S1 Fig Microscopy images of Δ*wetA* colonies under light and dark conditions. (PDF)

S2 Fig AFB1 production of WT and Δ*wetA* conidia by TLC analysis. (PDF)

S1 Table *Aspergillus* strains and oligonucleotides used in this study. (PDF)

S2 Table Top enriched GO categories of decreased mRNA levels in the Δ*wetA* conidia. (PDF)

S3 Table Top enriched GO categories of genes showing increased mRNA levels in the Δ*wetA* conidia. (PDF)

S4 Table Top 100 genes showing decreased mRNA levels in the Δ*wetA* conidia. (PDF)

S5 Table Top 100 genes showing increased mRNA levels in the Δ*wetA* conidia. (PDF)

S6 Table DEGs related to asexual development. (PDF)

S7 Table DEGs related to conidia maturation. (PDF)

S8 Table mRNA levels of secondary metabolic clustered genes in *A*. *flavus*. (PDF)

S9 Table Brief description of WetA-regulated genes associated with conidiation. (PDF)

S10 Table DEGs predicted to encode transcription factors. (PDF)
