# Supplementary figures and images for: WetA bridges cellular and chemical development in Aspergillus flavus
Source: PLoS One. 2017 Jun 28;12(6):e0179571. doi: 10.1371/journal.pone.0179571 (PMC5489174; doi:10.1371/journal.pone.0179571)

S1 Fig. Microscopy images of  $\Delta wetA$  colonies under light and dark conditions

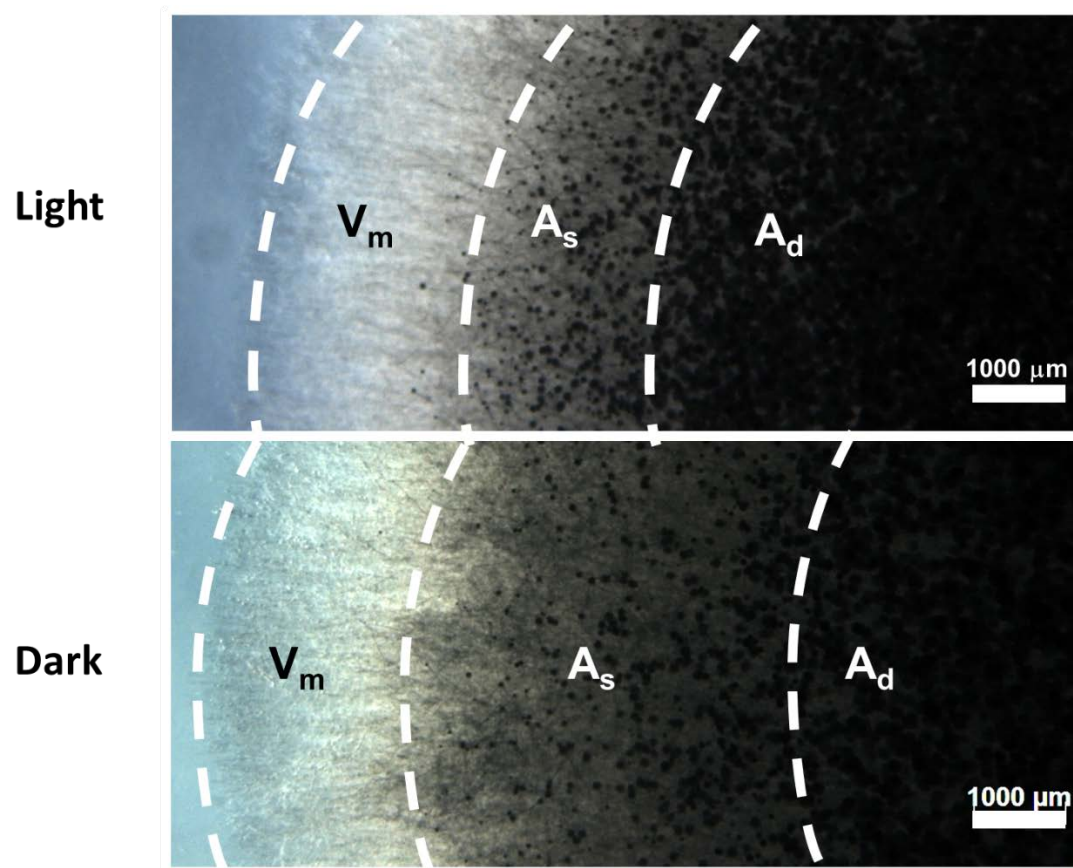

Supplement: S1 Fig — (PDF) [file pone.0179571.s001.pdf]

S2 Fig. AFB1 production of WT and  $\Delta wetA$  conidia by TLC analysis

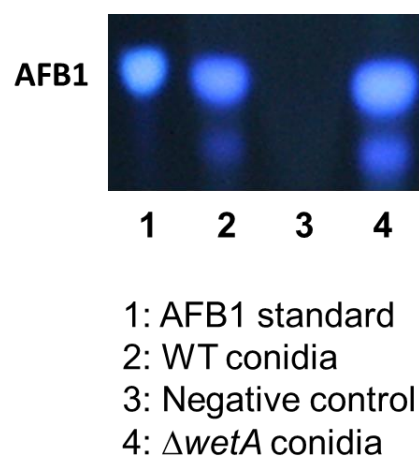

Supplement: S2 Fig — (PDF) [file pone.0179571.s002.pdf]
