# Supplementary material for: WetA bridges cellular and chemical development in Aspergillus flavus
Source: PLoS One. 2017 Jun 28;12(6):e0179571. doi: 10.1371/journal.pone.0179571 (PMC5489174; doi:10.1371/journal.pone.0179571)
Supplement: S1 Table — (PDF) [file pone.0179571.s003.pdf]

S1 Table. ***Aspergillus*** **strains and oligonucleotides used in this study.**

STRAINS		
**Name**	GENOTYPE	SOURCE
**NRRL3357**	Wild-type	Fungal Genetic Stock Center
**3357.5**	*pyrG*$^{-}$	[1]
**TMY1**	*ΔwetA::AfupyrG*$^{+}$*; pyrG*$^{-}$	This study
**TMY2**	*ΔwetA::AfupyrG*$^{+}$*; pyrG*$^{-}$*; wetA*	This study

OLIGONUCLEOTIDES		
**Name**	**SEQUENCE (5'→3')**	**PURPOSE**
**oMY-19**	ggagagcagattagcacctg	5' flanking of *wetA*
**oMY-20**	ttacctcgacatgccacag	3' flanking of *wetA*
**oMY-21**	ccatgcatggttactggagg	5' nested of *wetA*
**oMY-22**	atgcgaccacgaatgcatcc	3' nested of *wetA*
**oMY-51**	gggtgaagagcattgtttgaggcacgatggtggttccaagctcagctgtc	5' *wetA* with *AfupyrG* tail
**oMY-52**	tcagtgcctcctctcagacagaatatcggtgttctgatggatccggagtc	3' *wetA* with *AfupyrG* tail
**oMY-25**	gaccactcgttcaacaacgatg	5' *wetA*
**oMY-26**	cgtactgcattaagtgcgg	3' *wetA*
**oJH-84**	gctgaagtcatgatacaggccaaa	5' *AfupyrG* marker
**oJH-85**	atcgtcgggaggtattgtcgtcac	3' *AfupyrG* marker

1. He Z-M, Price MS, Obrian GR, Georgianna DR, Payne GA. Improved protocols for functional analysis in the pathogenic fungus Aspergillus flavus. BMC Microbiol. BioMed Central; 2007;7: 104. doi:10.1186/1471-2180-7-104
