# Supplementary material for: WetA bridges cellular and chemical development in Aspergillus flavus
Source: PLoS One. 2017 Jun 28;12(6):e0179571. doi: 10.1371/journal.pone.0179571 (PMC5489174; doi:10.1371/journal.pone.0179571)
Supplement: S2 Table — (PDF) [file pone.0179571.s004.pdf]

S2 Table. Top enriched GO categories of decreased mRNA levels in the Δ*wetA* conidia.

GO Category	# of Genes	% of Genes in Category
**Biological Process**		
metabolic process	846	25.22%
biosynthetic process	298	27.41%
organic substance biosynthetic process	280	27.78%
cellular biosynthetic process	275	28.09%
oxidation-reduction process	235	28.87%
cellular nitrogen compound biosynthetic process	209	33.07%
organonitrogen compound metabolic process	204	30.96%
organic cyclic compound biosynthetic process	161	28.75%
heterocycle biosynthetic process	157	30.13%
organonitrogen compound biosynthetic process	153	31.94%
cellular amide metabolic process	94	33.57%
amide biosynthetic process	86	33.46%
peptide metabolic process	73	33.64%
translation	60	38.22%
organophosphate metabolic process	60	34.29%
cofactor metabolic process	44	35.48%
nucleoside phosphate metabolic process	40	40.00%
coenzyme metabolic process	37	39.36%
pyridine-containing compound metabolic process	19	54.29%
pyridine nucleotide metabolic process	17	54.84%
nicotinamide nucleotide metabolic process	17	54.84%
vitamin metabolic process	16	66.67%
cellular modified amino acid metabolic process	14	51.85%
cellular modified amino acid biosynthetic process	13	59.09%
tetrapyrrole metabolic process	9	69.23%
GTP metabolic process	6	100.00%
molybdopterin cofactor metabolic process	6	85.71%
prosthetic group metabolic process	6	85.71%
**Molecular Function**		
oxidoreductase activity	1095	29.59%
RNA polymerase II transcription factor activity, sequence-specific DNA binding	167	35.33%
structural molecule activity	115	46.96%
structural constituent of ribosome	97	54.64%

Cellular Component		
extracellular region	74	29.60%
intracellular ribonucleoprotein complex	53	39.85%
ribonucleoprotein complex	53	39.85%
ribosome	53	54.64%
ribosomal subunit	12	63.16%
small ribosomal subunit	8	80.00%
