# Supplementary material for: WetA bridges cellular and chemical development in Aspergillus flavus
Source: PLoS One. 2017 Jun 28;12(6):e0179571. doi: 10.1371/journal.pone.0179571 (PMC5489174; doi:10.1371/journal.pone.0179571)
Supplement: S3 Table — (PDF) [file pone.0179571.s005.pdf]

S3 Table. Top enriched GO categories of genes showing increased mRNA levels in the Δ*wetA* conidia.

GO Category	# of Genes	% of Genes in Category
**Biological process**		
single-organism process	500	23.89%
transmembrane transport	167	25.97%
response to stimulus	113	29.66%
lipid metabolic process	86	28.67%
secondary metabolic process	77	35.48%
secondary metabolite biosynthetic process	73	35.27%
response to chemical	52	40.00%
cellular response to chemical stimulus	50	40.98%
cell wall organization or biogenesis	39	41.94%
asexual reproduction	38	34.86%
asexual sporulation	37	35.24%
toxin metabolic process	30	38.46%
response to drug	23	41.07%
cellular response to drug	22	41.51%
external encapsulating structure organization	21	48.84%
cell wall organization	21	48.84%
asexual sporulation resulting in formation of a cellular spore	21	42.00%
fungal-type cell wall organization or biogenesis	20	50.00%
chemical homeostasis	18	45.00%
aminoglycan metabolic process	15	57.69%
cation homeostasis	15	57.69%
ion homeostasis	15	51.72%
cell wall polysaccharide metabolic process	15	48.39%
glucosamine-containing compound metabolic process	14	66.67%
amino sugar metabolic process	14	60.87%
metal ion homeostasis	14	58.33%
inorganic ion homeostasis	14	51.85%
chitin metabolic process	11	61.11%
divalent inorganic cation homeostasis	8	66.67%
chitin biosynthetic process	6	85.71%
amino sugar biosynthetic process	6	85.71%
glucosamine-containing compound biosynthetic process	6	85.71%
positive regulation of conidiophore development	5	100.00%

**Molecular Function**		
catalytic activity	782	22.36%
iron ion binding	60	34.68%
flavin adenine dinucleotide binding	58	30.37%
tetrapyrrole binding	55	35.71%
heme binding	55	35.71%
oxidoreductase activity, acting on paired donors, with incorporation or reduction of molecular oxygen	55	32.96%
electron carrier activity	46	32.86%
monooxygenase activity	29	48.33%
acyl-CoA dehydrogenase activity	10	58.82%
chitin synthase activity	6	100.00%
1,3-beta-glucanosyltransferase activity	5	100.00%
**Cellular Component**		
membrane	277	26.01%
intrinsic component of membrane	173	25.48%
integral component of membrane	170	25.34%
extracellular region	80	32.00%
cell periphery	50	42.02%
plasma membrane	28	44.44%
cell septum	24	57.14%
site of polarized growth	23	50.00%
external encapsulating structure	22	52.38%
hyphal tip	22	50.00%
cell wall	21	53.85%
fungal-type cell wall	19	57.58%
plasma membrane part	10	55.56%
cell surface	9	81.82%
