# Supplementary material for: WetA bridges cellular and chemical development in Aspergillus flavus
Source: PLoS One. 2017 Jun 28;12(6):e0179571. doi: 10.1371/journal.pone.0179571 (PMC5489174; doi:10.1371/journal.pone.0179571)
Supplement: S4 Table — (PDF) [file pone.0179571.s006.pdf]

S4 Table. Top 100 genes showing decreased mRNA levels in the Δ*wetA* conidia.

GENE ID	Log_2 Fold Change	Description
**AFLA_126840**	-11.62	conserved hypothetical protein
**AFLA_071920**	-11.61	class III aminotransferase, putative
**AFLA_001980**	-11.56	glutamate cysteine ligase, putative
**AFLA_007360**	-11.52	hypothetical protein
**AFLA_116190**	-11.46	conserved hypothetical protein
**AFLA_102370**	-11.30	conserved hypothetical protein
**AFLA_124380**	-11.18	hypothetical protein
**AFLA_075640**	-11.09	pigment biosynthesis protein Ayg1
**AFLA_116180**	-11.04	hypothetical protein
**AFLA_120760**	-11.01	fatty acid oxygenase, putative
**AFLA_124110**	-10.99	galactose-proton symport, putative
**AFLA_096210**	-10.90	catalase, putative
**AFLA_077930**	-10.90	conserved hypothetical protein
**AFLA_126820**	-10.90	conserved hypothetical protein
**AFLA_004410**	-10.89	conserved hypothetical protein
**AFLA_066550**	-10.87	alcohol dehydrogenase, putative
**AFLA_117120**	-10.85	hypothetical protein
**AFLA_036400**	-10.80	conserved hypothetical protein
**AFLA_075630**	-10.79	conserved hypothetical protein
**AFLA_101770**	-10.79	conserved hypothetical protein
**AFLA_001030**	-10.79	lanosterol synthase, putative
**AFLA_007350**	-10.76	hypothetical protein
**AFLA_124890**	-10.74	hypothetical protein
**AFLA_052030**	-10.74	developmental regulatory protein WetA
**AFLA_056870**	-10.71	conserved hypothetical protein
**AFLA_124560**	-10.70	conserved hypothetical protein
**AFLA_126830**	-10.70	conserved hypothetical protein
**AFLA_064190**	-10.70	hypothetical protein
**AFLA_123950**	-10.69	ankyrin repeat protein
**AFLA_116170**	10.67	hypothetical protein
**AFLA_009740**	-10.66	hypothetical protein
**AFLA_023610**	-10.65	conserved hypothetical protein
**AFLA_126260**	-10.59	extracellular 3-ketosteroid 1-dehydrogenase, putative
**AFLA_034380**	-10.59	catalase, putative
**AFLA_007880**	-10.59	conserved hypothetical protein
**AFLA_085550**	-10.55	hypothetical protein
**AFLA_117110**	-10.50	conserved hypothetical protein
**AFLA_073970**	-10.48	conserved hypothetical protein

**AFLA_104490**	-10.46	conserved hypothetical protein
**AFLA_126170**	-10.41	conserved hypothetical protein
**AFLA_004440**	-10.38	ABC multidrug transporter, putative
**AFLA_101550**	-10.38	thioredoxin reductase, putative
**AFLA_072030**	-10.28	conserved hypothetical protein
**AFLA_063400**	-10.27	hypothetical protein
**AFLA_066540**	-10.24	hypothetical protein
**AFLA_096220**	-10.20	HHE domain protein
**AFLA_122950**	-10.17	conserved hypothetical protein
**AFLA_121110**	-10.13	conserved hypothetical protein
**AFLA_044800**	-10.11	conidiation protein Con-6, putative
**AFLA_000870**	-10.09	hypothetical protein
**AFLA_092300**	-10.09	hypothetical protein
**AFLA_063080**	-10.01	conserved hypothetical protein
**AFLA_063110**	-9.92	hypothetical protein
**AFLA_043550**	-9.85	conserved hypothetical protein
**AFLA_024660**	-9.83	conserved hypothetical protein
**AFLA_059700**	-9.82	conserved hypothetical protein
**AFLA_099050**	-9.79	conserved hypothetical protein
**AFLA_083110**	-9.72	conidiation-specific protein (Con-10), putative
**AFLA_034370**	-9.67	hypothetical protein
**AFLA_014240**	-9.66	conserved hypothetical protein
**AFLA_002840**	-9.66	HHE domain protein
**AFLA_117130**	-9.61	conserved hypothetical protein
**AFLA_003760**	-9.59	NACHT domain protein
**AFLA_059670**	-9.58	conserved hypothetical protein
**AFLA_008630**	-9.45	hypothetical protein
**AFLA_102410**	-9.44	conserved hypothetical protein
**AFLA_120190**	-9.42	67 kDa myosin-cross-reactive antigen family protein
**AFLA_077900**	-9.41	conserved hypothetical protein
**AFLA_057190**	-9.38	conserved hypothetical protein
**AFLA_033450**	-9.36	gamma-glutamylputrescine oxidoreductase, putative
**AFLA_010480**	-9.33	potassium channel, putative
**AFLA_007580**	-9.28	MFS sugar transporter, putative
**AFLA_102420**	-9.20	hypothetical protein
**AFLA_123310**	-9.17	conserved hypothetical protein
**AFLA_048440**	-9.13	conserved hypothetical protein
**AFLA_071780**	-9.06	Dyp-type peroxidase family protein
**AFLA_126860**	-9.03	cell wall cysteine-rich protein
**AFLA_059690**	-8.97	conserved hypothetical protein
**AFLA_039040**	-8.93	conserved hypothetical protein

**AFLA_131930**	-8.86	conserved hypothetical protein
**AFLA_090360**	-8.86	conserved hypothetical protein
**AFLA_106210**	-8.85	efflux pump antibiotic resistance protein, putative
**AFLA_010090**	-8.85	hypothetical protein
**AFLA_096740**	-8.85	monoxygenase, putative
**AFLA_008410**	-8.81	MFS multidrug transporter, putative
**AFLA_074740**	-8.77	conserved hypothetical protein
**AFLA_039050**	-8.71	hypothetical protein
**AFLA_131600**	-8.69	lysine-rich arabinogalactan protein 18 precursor, putative
**AFLA_092090**	-8.67	oxidoreductase, short-chain dehydrogenase/reductase family
**AFLA_016440**	-8.67	conserved hypothetical protein
**AFLA_124370**	-8.64	hypothetical protein
**AFLA_126850**	-8.63	vacuolar protease A, putative
**AFLA_094770**	-8.62	conserved hypothetical protein
**AFLA_024540**	-8.52	sensory transduction histidine kinase, putative
**AFLA_015260**	-8.50	short chain dehydrogenase/oxidoreductase, putative
**AFLA_106220**	-8.50	cytochrome P450, putative
**AFLA_072380**	-8.50	conserved hypothetical protein
**AFLA_024610**	-8.49	NADP-dependent alcohol dehydrogenase
**AFLA_053400**	-8.47	hypothetical protein
**AFLA_120210**	-8.44	conserved hypothetical protein

-
