# Supplementary material for: WetA bridges cellular and chemical development in Aspergillus flavus
Source: PLoS One. 2017 Jun 28;12(6):e0179571. doi: 10.1371/journal.pone.0179571 (PMC5489174; doi:10.1371/journal.pone.0179571)
Supplement: S5 Table — (PDF) [file pone.0179571.s007.pdf]

S5 Table. Top 100 genes showing increased mRNA levels in the Δ*wetA* conidia.

GENE ID	Log_2 Fold Change	Description
**AFLA_121740**	10.28	hypothetical protein
**AFLA_070470**	8.84	conserved hypothetical protein
**AFLA_118990**	7.89	efflux pump antibiotic resistance protein, putative
**AFLA_020010**	7.69	hypothetical protein
**AFLA_106850**	7.56	thioredoxin reductase GliT-like, putative
**AFLA_059770**	7.54	conserved hypothetical protein
**AFLA_064950**	7.51	conserved hypothetical protein
**AFLA_106040**	7.48	conserved hypothetical protein
**AFLA_060200**	7.45	neutral amino acid permease, putative
**AFLA_135310**	7.43	hypothetical protein
**AFLA_045570**	7.40	acetyl xylan esterase, putative
**AFLA_134850**	7.37	conserved hypothetical protein
**AFLA_059950**	7.37	oxidoreductase, FAD-binding, putative
**AFLA_033410**	7.37	conserved hypothetical protein
**AFLA_116630**	7.35	(S)-2-hydroxy-acid oxidase, putative
**AFLA_064840**	7.27	conserved hypothetical protein
**AFLA_059900**	7.21	calcium-binding protein precursor, putative
**AFLA_004880**	7.10	amine oxidase, flavin-containing superfamily
**AFLA_100200**	7.01	conserved hypothetical protein
**AFLA_001750**	6.90	cell surface protein Mas1, putative
**AFLA_073230**	6.83	conserved hypothetical protein
**AFLA_064850**	6.83	aminotriazole resistance protein, putative
**AFLA_107900**	6.82	conserved hypothetical protein
**AFLA_104680**	6.80	class V chitinase ChiB1
**AFLA_137110**	6.79	extracellular serine-rich protein, putative
**AFLA_043870**	6.71	conserved hypothetical protein
**AFLA_104880**	6.68	glycosyl hydrolase, putative
**AFLA_033420**	6.61	Mn superoxide dismutase MnSOD
**AFLA_060340**	6.56	conserved hypothetical protein
**AFLA_052490**	6.55	C2H2 finger domain protein, putative
**AFLA_135300**	6.54	hypothetical protein
**AFLA_073220**	6.51	hypothetical protein
**AFLA_008450**	6.50	conserved hypothetical protein
**AFLA_087030**	6.41	conserved hypothetical protein
**AFLA_004510**	6.39	peroxidase, putative
**AFLA_125710**	6.37	conserved hypothetical protein
**AFLA_065460**	6.36	conserved hypothetical protein

**AFLA_137940**	6.36	conserved hypothetical protein
**AFLA_138180**	6.34	lysozyme, putative
**AFLA_085820**	6.32	conserved hypothetical protein
**AFLA_129170**	6.30	conserved hypothetical protein
**AFLA_106070**	6.30	conserved hypothetical protein
**AFLA_123580**	6.28	hypothetical protein
**AFLA_023760**	6.28	conserved hypothetical protein
**AFLA_061710**	6.15	calcium/calmodulin-dependent protein kinase type, putative
**AFLA_040090**	6.14	conserved hypothetical protein
**AFLA_053300**	6.10	cytochrome P450, putative
**AFLA_102200**	6.09	conserved hypothetical protein
**AFLA_120930**	6.03	glycosyl transferase, putative
**AFLA_131620**	6.00	acyl-CoA desaturase, putative
**AFLA_041690**	5.98	cysteine synthase B, putative
**AFLA_053560**	5.90	conserved hypothetical protein
**AFLA_060010**	5.88	PKS-like enzyme, putative
**AFLA_065760**	5.87	cytochrome P450, putative
**AFLA_001680**	5.87	conserved hypothetical protein
**AFLA_129490**	5.86	hypothetical protein
**AFLA_082410**	5.84	conserved hypothetical protein
**AFLA_122200**	5.83	conserved hypothetical protein
**AFLA_099940**	5.82	conserved hypothetical protein
**AFLA_070320**	5.80	choline transport protein, putative
**AFLA_031380**	5.80	class V chitinase, putative
**AFLA_039740**	5.80	conserved hypothetical protein
**AFLA_005370**	5.80	conserved hypothetical protein
**AFLA_067760**	5.78	conserved histidine-rich protein
**AFLA_059610**	5.68	conserved hypothetical protein
**AFLA_100210**	5.66	ankyrin repeat-containing protein, putative
**AFLA_137860**	5.64	conserved hypothetical protein
**AFLA_117000**	5.64	RNA exonuclease, putative
**AFLA_072810**	5.62	conserved hypothetical protein
**AFLA_110980**	5.62	conserved hypothetical protein
**AFLA_045580**	5.61	hypothetical protein
**AFLA_029970**	5.60	conserved hypothetical protein
**AFLA_116260**	5.58	N-hydroxyarylamine O-acetyltransferase, putative
**AFLA_034810**	5.56	hypothetical protein
**AFLA_023250**	5.53	conserved hypothetical protein
**AFLA_023160**	5.53	ankyrin repeat-rich membrane-spanning protein, putative
**AFLA_060110**	5.52	conserved hypothetical protein

**AFLA_001670**	5.52	3-demethylubiquinone-9 3-methyltransferase, putative
**AFLA_066790**	5.51	conserved hypothetical protein
**AFLA_137850**	5.50	conserved hypothetical protein
**AFLA_019000**	5.49	conserved hypothetical protein
**AFLA_031140**	5.49	LipA and NB-ARC domain protein
**AFLA_074240**	5.48	conserved hypothetical protein
**AFLA_075190**	5.48	conserved hypothetical protein
**AFLA_064810**	5.46	extracellular proline-serine rich protein
**AFLA_095440**	5.45	serine/threonine protein kinase, putative
**AFLA_010870**	5.43	endo-1,4-beta-xylanase B precursor, putative
**AFLA_097190**	5.42	conserved hypothetical protein
**AFLA_009870**	5.41	conserved hypothetical protein
**AFLA_134330**	5.41	conserved hypothetical protein
**AFLA_065050**	5.39	Defensin domain protein
**AFLA_077720**	5.37	conserved hypothetical protein
**AFLA_077730**	5.36	carnitinyl-CoA dehydratase, putative
**AFLA_101360**	5.36	cytochrome P450, putative
**AFLA_107340**	5.35	C4-dicarboxylate transporter/malic acid transport protein, putative
**AFLA_078910**	5.35	hypothetical protein
**AFLA_101190**	5.33	conserved hypothetical protein
**AFLA_034670**	5.31	poly(ADP)-ribose polymerase PARP, putative
**AFLA_121660**	5.30	conserved hypothetical protein
**AFLA_042430**	5.28	hypothetical protein
