# Supplementary material for: WetA bridges cellular and chemical development in Aspergillus flavus
Source: PLoS One. 2017 Jun 28;12(6):e0179571. doi: 10.1371/journal.pone.0179571 (PMC5489174; doi:10.1371/journal.pone.0179571)
Supplement: S6 Table — (PDF) [file pone.0179571.s008.pdf]

S6 Table. DEGs related to asexual development.

Gene ID	Log2 Fold Change	Common Name
**AFLA_034670**	5.31	*prpA*
**AFLA_033400**	4.17	*msdS*
**AFLA_021100**	3.74	*ppoD*
**AFLA_099380**	3.60	*wsc1*
**AFLA_136030**	3.37	*chsE*
**AFLA_082850**	3.31	*brlA*
**AFLA_137320**	3.04	*flbC*
**AFLA_052510**	3.01	*wsc3*
**AFLA_029620**	2.96	*abaA*
**AFLA_008970**	2.89	*llmF*
**AFLA_031700**	2.89	*midA*
**AFLA_087350**	2.86	*sltA*
**AFLA_136410**	2.81	*medA*
**AFLA_131330**	2.72	*nsdC*
**AFLA_060590**	2.59	*chsG*
**AFLA_071090**	2.51	*esdC*
**AFLA_028750**	2.49	*llmB*
**AFLA_078290**	2.43	*chsF*
**AFLA_029150**	2.40	*pcl1*
**AFLA_086010**	2.40	*figA*
**AFLA_135550**	2.38	*mob1*
**AFLA_046990**	2.32	*stuA*
**AFLA_091490**	2.27	*mtfA*
**AFLA_102850**	2.22	*rgdA*
**AFLA_092800**	2.17	*nudA*
**AFLA_042780**	1.95	*chsA*
**AFLA_066330**	1.85	*odeA*
**AFLA_083100**	1.81	*zipA*
**AFLA_019100**	1.74	*fbx15*
**AFLA_098380**	1.72	*rodA*
**AFLA_114760**	1.72	*chsB*
**AFLA_134030**	1.71	*flbA*
**AFLA_136540**	1.71	*ime2*
**AFLA_020210**	1.68	*nsdD*
**AFLA_093230**	1.66	*phnA*
**AFLA_037320**	1.58	*rho1*

**AFLA_127920**	1.57	*crzA*
**AFLA_068440**	1.53	*cch1*
**AFLA_132340**	1.48	*vapA*
**AFLA_048880**	1.48	*steC*
**AFLA_004760**	1.40	*ssc1*
**AFLA_091740**	1.39	*sidB*
**AFLA_110620**	1.39	*atg1*
**AFLA_020990**	1.34	*ugtA*
**AFLA_030430**	1.23	*ppoC*
**AFLA_022400**	1.23	*atgH*
**AFLA_085200**	1.08	*pac2/osaB*
**AFLA_086900**	1.02	*ams1*
**AFLA_028410**	-1.02	*pbcR*
**AFLA_088670**	-1.05	*kex1*
**AFLA_110790**	-1.07	*ricA*
**AFLA_082510**	-1.07	*tcpA*
**AFLA_065850**	-1.09	*fphA*
**AFLA_114720**	-1.12	*bem1*
**AFLA_048000**	-1.15	*rhbA*
**AFLA_029640**	-1.26	*nudG*
**AFLA_032870**	-1.36	*pkaR*
**AFLA_044820**	-1.43	*swoM*
**AFLA_026790**	-1.46	*ppoA*
**AFLA_055650**	-1.50	*osaA*
**AFLA_112660**	-1.53	*argB*
**AFLA_066460**	-1.90	*veA*
**AFLA_014260**	-2.00	*rodB*
**AFLA_091910**	-2.09	*pkaB*
**AFLA_131370**	-2.30	*tpsC*
**AFLA_093580**	-2.48	*tmpA*
**AFLA_039530**	-2.59	*fluG*
**AFLA_135040**	-2.63	*pkaA*
**AFLA_087630**	-2.65	*tpsA* ortholog
**AFLA_005520**	-2.82	*sfgA*
**AFLA_026900**	-3.70	*vosA*
**AFLA_060780**	-4.32	*dewA*
**AFLA_062460**	-5.13	*nce102*
**AFLA_006170**	-5.70	*wA/pksP*
**AFLA_074470**	-6.25	*rft1*

**AFLA_052030**	-10.74	*wetA*
**AFLA_120760**	-11.01	*ppoB*
