# Supplementary material for: WetA bridges cellular and chemical development in Aspergillus flavus
Source: PLoS One. 2017 Jun 28;12(6):e0179571. doi: 10.1371/journal.pone.0179571 (PMC5489174; doi:10.1371/journal.pone.0179571)
Supplement: S7 Table — (PDF) [file pone.0179571.s009.pdf]

S7 Table. DEGs related to conidia maturation.

Gene ID	Log_2 Fold Change	Common Name/ Function
**AFLA_075640**	-11.09	*ayg1*
**AFLA_063080**	-10.01	putative hydrophobin
**AFLA_006170**	-5.70	*pksP*
**AFLA_060780**	-4.32	*dewA*
**AFLA_023650**	-4.21	endo-1,3-1,4-beta-D-glucanase, putative
**AFLA_002830**	-3.85	*tpsc*
**AFLA_028260**	-3.06	*exg1*
**AFLA_090490**	-2.85	*treA*
**AFLA_087630**	-2.65	alpha,alpha-trehalose-phosphate synthase subunit, putative
**AFLA_068300**	-2.36	*bgt1*
**AFLA_131370**	-2.30	*tppb*
**AFLA_098980**	-2.28	putative hydrophobin
**AFLA_030450**	-2.24	*ccg-9*
**AFLA_008180**	-2.02	*rfab*
**AFLA_014260**	-2.00	*rodB*
**AFLA_077910**	-1.86	*agnD*
**AFLA_023460**	-1.76	*ags1*
**AFLA_087640**	-1.09	*tppc*
**AFLA_004480**	1.17	*eng4*
**AFLA_129100**	1.37	*exg2*
**AFLA_091790**	1.43	*agnE*
**AFLA_091790**	1.43	alpha-1,3-glucanase/mutanase, putative
**AFLA_098380**	1.72	*rodA*
**AFLA_108860**	1.92	*gel2*
**AFLA_052800**	1.92	*fksP*
**AFLA_042780**	1.95	*chsA*
**AFLA_006590**	2.02	*chiA*
**AFLA_107830**	2.03	brain chitinase and chia, putative
**AFLA_107790**	2.09	glucan 1,3-beta-glucosidase precursor, putative
**AFLA_095890**	2.10	*eng8*
**AFLA_033550**	2.30	*cts2*
**AFLA_078290**	2.43	*chesF*
**AFLA_121370**	2.45	*gel4*
**AFLA_060590**	2.59	*chsG*
**AFLA_058480**	2.67	*gel1*

**AFLA_052810**	2.72	*gel7*
**AFLA_041950**	2.77	*exg0*
**AFLA_064920**	2.81	1,3-beta-glucanosyltransferase *gel4* precursor, putative
**AFLA_078900**	2.97	*nagA*
**AFLA_129440**	3.02	*gel5*
**AFLA_057680**	3.18	beta-N-hexosaminidase, putative
**AFLA_136030**	3.37	*chsE*
**AFLA_020630**	3.56	*gel6*
**AFLA_028950**	3.61	*engl1*
**AFLA_136040**	3.86	*chsZ*
**AFLA_013690**	3.97	*chsC*
**AFLA_101800**	4.16	*ctcB*
**AFLA_134100**	4.21	*ags2*
**AFLA_094600**	4.57	putative hydrophobin
**AFLA_029950**	5.25	*eng3*
**AFLA_031380**	5.80	class V chitinase, putative
**AFLA_104680**	6.80	*chiB*
