# Supplementary material for: WetA bridges cellular and chemical development in Aspergillus flavus
Source: PLoS One. 2017 Jun 28;12(6):e0179571. doi: 10.1371/journal.pone.0179571 (PMC5489174; doi:10.1371/journal.pone.0179571)
Supplement: S8 Table — (PDF) [file pone.0179571.s010.pdf]

S8 Table. mRNA levels of secondary metabolic clustered genes in *A. flavus*.

Cluster	Gene ID	Log_2 Fold Change	Description
**1**	**AFLA_125780**	-	ATP-binding cassette transporter, putative
**1**	**AFLA_125770**	-1.76	LysR family regulatory protein, putative
**1**	**AFLA_125760**	-1.24	squalene-hopene-cyclase, putative
**2**	**AFLA_126710**	-	polyketide synthase, putative
**2**	**AFLA_126720**	-	hypothetical protein
**2**	**AFLA_126730**	-	conserved hypothetical protein
**2**	**AFLA_126740**	-	lipase precursor, putative
**3**	**AFLA_126970**	-	arginine permease, putative
**3**	**AFLA_126980**	-	conserved hypothetical protein
**3**	**AFLA_126990**	-	conserved hypothetical protein
**3**	**AFLA_127000**	-	hypothetical protein
**3**	**AFLA_127010**	-	conserved hypothetical protein
**3**	**AFLA_127020**	-	monooxygenase, putative
**3**	**AFLA_127030**	-	conserved hypothetical protein
**3**	**AFLA_127040**	-	MFS monocarboxylate transporter, putative
**3**	**AFLA_127050**	-	conserved hypothetical protein
**3**	**AFLA_127060**	-	conserved hypothetical protein
**3**	**AFLA_127070**	-	short-chain dehydrogenase, putative
**3**	**AFLA_127080**	-	conserved hypothetical protein
**3**	**AFLA_127100**	-	conserved hypothetical protein
**3**	**AFLA_127110**	-	MFS transporter, putative
**3**	**AFLA_127120**	-	hypothetical protein
**3**	**AFLA_127130**	-	conserved hypothetical protein
**3**	**AFLA_127140**	-	conserved hypothetical protein
**3**	**AFLA_127150**	-	hypothetical protein
**3**	**AFLA_127160**	-	NB-ARC and TPR domain protein
**3**	**AFLA_127170**	-	penicillin-binding protein, putative
**3**	**AFLA_127090**	-2.42	polyketide synthase, putative
**4**	**AFLA_128040**	-	efflux pump antibiotic resistance protein, putative
**4**	**AFLA_128060**	-	polyketide synthase, putative
**4**	**AFLA_128050**	1.12	conserved hypothetical protein
**5**	**AFLA_128160**	-	C6 transcription factor, putative
**5**	**AFLA_128170**	-	NRPS-like enzyme, putative
**6**	**AFLA_053900**	-	conserved hypothetical protein
**6**	**AFLA_053910**	-	pantothenate transporter, putative

**6**	**AFLA_053950**	-	conserved hypothetical protein
**6**	**AFLA_053880**	-2.39	hypothetical protein
**6**	**AFLA_053930**	-2.24	multicopper oxidase, putative
**6**	**AFLA_053940**	-1.99	multicopper oxidase, putative
**6**	**AFLA_053890**	-1.89	acetylcholinesterase, putative
**6**	**AFLA_053870**	1.99	polyketide synthase, putative
**6**	**AFLA_053920**	3.88	hypothetical protein
**7**	**AFLA_054040**	-	conserved hypothetical protein
**7**	**AFLA_054070**	-	conserved hypothetical protein
**7**	**AFLA_054090**	-	polyketide synthase, putative
**7**	**AFLA_054100**	-	DUF341 family oxidoreductase, putative
**7**	**AFLA_054080**	-2.74	Na(+)/H(+) antiporter, putative
**7**	**AFLA_054050**	1.24	ABC multidrug transporter, putative
**7**	**AFLA_054060**	5.22	ATP/GTP-binding protein, putative
**8**	**AFLA_054180**	-	conserved hypothetical protein
**8**	**AFLA_054190**	-	conserved hypothetical protein
**8**	**AFLA_054200**	-	hypothetical protein
**8**	**AFLA_054210**	-	ankyrin repeat-containing protein, putative
**8**	**AFLA_054220**	-	conserved hypothetical protein
**8**	**AFLA_054230**	-	conserved hypothetical protein
**8**	**AFLA_054250**	-	conserved hypothetical protein
**8**	**AFLA_054260**	-	MFS transporter, putative
**8**	**AFLA_054270**	-	NRPS-like enzyme, putative
**8**	**AFLA_054280**	-	conserved hypothetical protein
**8**	**AFLA_054290**	-	aldehyde reductase I (ARI), putative
**8**	**AFLA_054300**	-	pantothenate transporter, putative
**8**	**AFLA_054320**	-	NPP1 domain protein, putative
**8**	**AFLA_054370**	-3.48	short-chain dehydrogenase, putative
**8**	**AFLA_054310**	-3.24	conserved hypothetical protein
**8**	**AFLA_054330**	-2.68	conserved hypothetical protein
**8**	**AFLA_054340**	-1.28	isoflavone reductase family protein
**8**	**AFLA_054350**	-1.27	actin-binding protein Fragmin, putative
**8**	**AFLA_054240**	2.53	conserved hypothetical protein
**8**	**AFLA_054360**	3.36	conserved hypothetical protein
**9**	**AFLA_008700**	-	monooxygenase, putative
**9**	**AFLA_008710**	-	monooxygenase, putative
**9**	**AFLA_008740**	-	oxidoreductase, short chain dehydrogenase/reductase family
**9**	**AFLA_008760**	-	multidrug resistance protein, putative

**9**	**AFLA_008770**	-	nonribosomal peptide synthase, putative
**9**	**AFLA_008780**	-	asparagine synthetase, putative
**9**	**AFLA_008790**	-	conserved hypothetical protein
**9**	**AFLA_008730**	-3.17	hypothetical protein
**9**	**AFLA_008720**	-1.54	conserved hypothetical protein
**9**	**AFLA_008750**	3.63	hypothetical protein
**10**	**AFLA_009980**	-	ABC multidrug transporter, putative
**10**	**AFLA_010030**	-	oxidoreductase, 2OG-Fe(II) oxygenase family, putative
**10**	**AFLA_010040**	-	conserved hypothetical protein
**10**	**AFLA_010050**	-3.13	alcohol dehydrogenase, putative
**10**	**AFLA_010020**	-1.84	nonribosomal peptide synthase, putative
**10**	**AFLA_010010**	-1.18	nonribosomal peptide synthase, putative
**10**	**AFLA_010000**	1.98	polyketide synthase, putative
**10**	**AFLA_009990**	3.05	1-aminocyclopropane-1-carboxylate oxidase, putative
**11**	**AFLA_010590**	-	siderophore biosynthesis lipase/esterase, putative
**11**	**AFLA_010600**	-	siderophore biosynthesis acetylase AceI, putative
**11**	**AFLA_010610**	-	enoyl-CoA hydratase/isomerase family protein
**11**	**AFLA_010640**	-2.52	MFS siderophore transporter, putative
**11**	**AFLA_010630**	-2.47	ABC multidrug transporter SidT
**11**	**AFLA_010620**	1.56	nonribosomal siderophore peptide synthase Sid2
**12**	**AFLA_079370**	-	hypothetical protein
**12**	**AFLA_079380**	-	NRPS-like enzyme, putative
**12**	**AFLA_079390**	-	hypothetical protein
**12**	**AFLA_079400**	-	NRPS-like enzyme, putative
**12**	**AFLA_079420**	-	conserved hypothetical protein
**12**	**AFLA_079440**	-	allantoate permease, putative
**12**	**AFLA_079460**	-	DNA mismatch repair protein Msh1, putative
**12**	**AFLA_079360**	-4.14	PKS-like enzyme, putative
**12**	**AFLA_079430**	-3.82	amidase, putative
**12**	**AFLA_079450**	-2.39	ureidoglycolate hydrolase, putative
**12**	**AFLA_079410**	-2.04	MFS transporter, putative
**13**	**AFLA_080490**	-	polyketide synthase, putative
**13**	**AFLA_080510**	-	conserved hypothetical protein
**13**	**AFLA_080550**	-	conserved hypothetical protein
**13**	**AFLA_080500**	-1.47	cation-transporting ATPase, putative
**13**	**AFLA_080480**	1.10	HD superfamily hydrolase, putative
**13**	**AFLA_080520**	1.46	ADP-ribosylglycohydrolase family protein
**13**	**AFLA_080540**	1.98	DEAD box helicase Mph1, putative
**13**	**AFLA_080470**	3.03	conserved hypothetical protein

**13**	**AFLA_080530**	3.07	conserved hypothetical protein
**14**	**AFLA_082050**	-	epimerase/dehydratase family protein, putative
**14**	**AFLA_082060**	-	conserved hypothetical protein
**14**	**AFLA_082070**	-	pantothenate transporter, putative
**14**	**AFLA_082080**	-	alpha/beta hydrolase, putative
**14**	**AFLA_082090**	-	conserved hypothetical protein
**14**	**AFLA_082100**	-	conserved hypothetical protein
**15**	**AFLA_082150**	-	polyketide synthase, putative
**15**	**AFLA_082170**	-	efflux pump antibiotic resistance protein, putative
**15**	**AFLA_082180**	-	hypothetical protein
**15**	**AFLA_082190**	-	conserved hypothetical protein
**15**	**AFLA_082210**	1.94	conserved hypothetical protein
**15**	**AFLA_082230**	2.05	pantothenate transporter, putative
**15**	**AFLA_082160**	2.47	MFS glucose transporter, putative
**15**	**AFLA_082220**	2.70	short-chain dehydrogenase, putative
**15**	**AFLA_082200**	4.14	AAA family ATPase, putative
**16**	**AFLA_082430**	-	conserved hypothetical protein
**16**	**AFLA_082440**	-	conserved hypothetical protein
**16**	**AFLA_082480**	-	NRPS-like enzyme, putative
**16**	**AFLA_082470**	2.61	AMP dependent CoA ligase, putative
**16**	**AFLA_082450**	3.29	bicyclomycin resistance protein, putative
**16**	**AFLA_082460**	4.47	conserved hypothetical protein
**17**	**AFLA_083210**	-	conserved hypothetical protein
**17**	**AFLA_083220**	-	ATP dependent RNA helicase (Rok1), putative
**17**	**AFLA_083230**	-	mitochondrial F1F0-ATP synthase g subunit, putative
**17**	**AFLA_083260**	-	hypothetical protein
**17**	**AFLA_083270**	2.12	GABA permease, putative
**17**	**AFLA_083250**	2.43	dimethylallyl tryptophan synthase, putative
**17**	**AFLA_083240**	3.57	xylitol dehydrogenase LadA/XdhB
**18**	**AFLA_087830**	-	DNA excision repair protein Rad2
**18**	**AFLA_087850**	-	conserved hypothetical protein
**18**	**AFLA_087820**	-3.25	cell wall integrity signaling protein Lsp1/Pil1, putative
**18**	**AFLA_087840**	-1.43	WD repeat protein
**18**	**AFLA_087810**	-1.07	bZIP transcription factor, putative
**18**	**AFLA_087860**	1.80	cellulose-binding GDSL lipase/acylhydrolase, putative
**18**	**AFLA_087870**	3.02	endoglucanase, putative
**18**	**AFLA_087880**	5.24	integral membrane protein
**19**	**AFLA_114820**	-	polyketide synthase, putative
**19**	**AFLA_114830**	-	aminopeptidase, putative

**19**	**AFLA_114840**	-	conserved hypothetical protein
**20**	**AFLA_116140**	-	putative secreted protein
**20**	**AFLA_116230**	-	conserved hypothetical protein
**20**	**AFLA_116300**	-	short-chain dehydrogenase, putative
**20**	**AFLA_116310**	-	short-chain dehydrogenase, putative
**20**	**AFLA_116330**	-	O-methyltransferase, putative
**20**	**AFLA_116190**	-11.46	conserved hypothetical protein
**20**	**AFLA_116180**	-11.04	hypothetical protein
**20**	**AFLA_116170**	-10.67	hypothetical protein
**20**	**AFLA_116200**	-8.39	conserved hypothetical protein
**20**	**AFLA_116210**	-4.69	O-methyltransferase, putative
**20**	**AFLA_116290**	-3.47	conserved hypothetical protein
**20**	**AFLA_116280**	-3.36	dihydroxy-acid dehydratase, putative
**20**	**AFLA_116270**	-3.06	paraoxonase, putative
**20**	**AFLA_116220**	-3.01	polyketide synthase, putative
**20**	**AFLA_116320**	-1.97	hypothetical protein
**20**	**AFLA_116150**	-1.67	integral membrane protein
**20**	**AFLA_116160**	1.32	zinc-binding dehydrogenase family oxidoreductase
**20**	**AFLA_116250**	1.44	conserved hypothetical protein
**20**	**AFLA_116240**	4.52	hypothetical protein
**20**	**AFLA_116260**	5.58	N-hydroxyarylamine O-acetyltransferase, putative
**21**	**AFLA_116840**	-	salicylate 1-monooxygenase SalA
**21**	**AFLA_116850**	-	Ankyrin domain protein
**21**	**AFLA_116880**	-	C6 transcription factor, putative
**21**	**AFLA_116900**	-	conserved hypothetical protein
**21**	**AFLA_116860**	-5.73	alpha-N-acetylglucosaminidase, putative
**21**	**AFLA_116870**	-2.05	transferase family protein
**21**	**AFLA_116830**	-1.28	hypothetical protein
**21**	**AFLA_116890**	-1.02	polyketide synthase, putative
**22**	**AFLA_117770**	-	DnaJ domain protein
**22**	**AFLA_117780**	-	farnesyl-diphosphate farnesyltransferase, putative
**22**	**AFLA_117760**	2.10	phytase, putative
**23**	**AFLA_118440**	-4.87	NRPS-like enzyme, putative
**23**	**AFLA_118430**	-3.82	conserved hypothetical protein
**24**	**AFLA_118820**	-	short-chain dehydrogenase, putative
**24**	**AFLA_118830**	-	conserved hypothetical protein
**24**	**AFLA_118860**	-	hypothetical protein
**24**	**AFLA_118870**	-	translation initiation inhibitor, putative
**24**	**AFLA_118880**	-	gibberellin 3-beta hydroxylase, putative

**24**	**AFLA_118890**	-	eukaryotic translation initiation factor eIF-5A-2
**24**	**AFLA_118900**	-	conserved hypothetical protein
**24**	**AFLA_118910**	-	conserved hypothetical protein
**24**	**AFLA_118920**	-	hypothetical protein
**24**	**AFLA_118930**	-	hypothetical protein
**24**	**AFLA_118980**	-	mitochondrial carrier protein Leu5
**24**	**AFLA_118840**	-4.41	pantothenate transporter, putative
**24**	**AFLA_118850**	-3.61	2,4-dihydroxyhept-2-ene-1,7-dioic acid aldolase, putative
**24**	**AFLA_118940**	1.18	polyketide synthase, putative
**24**	**AFLA_118960**	2.48	polyketide synthase, putative
**24**	**AFLA_118970**	3.45	MAK1-like monooxygenase, putative
**24**	**AFLA_118950**	4.67	conserved hypothetical protein
**24**	**AFLA_118990**	7.89	efflux pump antibiotic resistance protein, putative
**25**	**AFLA_119100**	-	hypothetical protein
**25**	**AFLA_119090**	-3.44	oxidoreductase, short chain dehydrogenase/reductase family, putative
**25**	**AFLA_119120**	-3.25	beta-lactamase family protein
**25**	**AFLA_119110**	-1.86	NRPS-like enzyme, putative
**25**	**AFLA_119080**	-1.66	hypothetical protein
**26**	**AFLA_119750**	-	riboflavin aldehyde-forming enzyme
**26**	**AFLA_119790**	-	beta-xylosidase
**26**	**AFLA_119810**	-	sensory transduction histidine kinase bacterial, putative
**26**	**AFLA_119860**	-	pectin lyase, putative
**26**	**AFLA_119870**	-	iron-sulfur protein subunit of succinate dehydrogenase Sdh2, putative
**26**	**AFLA_119850**	-2.57	LsdA family protein
**26**	**AFLA_119770**	-2.49	conserved hypothetical protein
**26**	**AFLA_119780**	-2.14	neutral protease 2 precursor, putative
**26**	**AFLA_119880**	-1.51	hypothetical protein
**26**	**AFLA_119840**	-1.45	hypothetical protein
**26**	**AFLA_119760**	-1.13	conserved hypothetical protein
**26**	**AFLA_119800**	1.00	conserved hypothetical protein
**26**	**AFLA_119820**	1.59	NRPS-like enzyme, putative
**26**	**AFLA_119830**	2.66	MFS multidrug transporter, putative
**27**	**AFLA_121400**	-	hypothetical protein
**27**	**AFLA_121410**	-	hypothetical protein
**27**	**AFLA_121420**	-	hypothetical protein
**27**	**AFLA_121430**	-	conserved hypothetical protein
**27**	**AFLA_121440**	-	fumarylacetoacetate hydrolase, putative

**27**	**AFLA_121460**	-	hypothetical protein
**27**	**AFLA_121470**	-	hscarg dehydrogenase, putative
**27**	**AFLA_121540**	-	MFS multidrug transporter, putative
**27**	**AFLA_121580**	-	C-4 methyl sterol oxidase, putative
**27**	**AFLA_121590**	-3.55	zinc-binding alcohol dehydrogenase, putative
**27**	**AFLA_121450**	-2.35	trihydroxytoluene oxygenase
**27**	**AFLA_121600**	-1.73	conserved hypothetical protein
**27**	**AFLA_121550**	1.94	conserved hypothetical protein
**27**	**AFLA_121560**	1.97	conserved hypothetical protein
**27**	**AFLA_121570**	2.40	conserved hypothetical protein
**27**	**AFLA_121520**	2.54	NRPS-like enzyme, putative
**27**	**AFLA_121500**	2.78	cytochrome P450, putative
**27**	**AFLA_121490**	2.81	NmrA-like family protein
**27**	**AFLA_121530**	2.87	NADH-dependent flavin oxidoreductase, putative
**27**	**AFLA_121480**	3.14	phytanoyl-CoA dioxygenase family protein
**27**	**AFLA_121510**	3.53	conserved hypothetical protein
**28**	**AFLA_089620**	-	chalcone synthase, putative
**28**	**AFLA_089630**	-	choline transport protein, putative
**28**	**AFLA_089640**	-	conserved hypothetical protein
**28**	**AFLA_089650**	-	high-affinity iron transporter FtrA
**28**	**AFLA_089660**	-	ferrooxidoreductase Fet3, putative
**28**	**AFLA_089670**	-3.14	ferric reductase transmembrane component 4 precursor, putative
**29**	**AFLA_090190**	-	dimethylallyl tryptophan synthase, putative
**29**	**AFLA_090200**	-	nonribosomal peptide synthase, putative
**29**	**AFLA_090180**	3.80	alpha/beta hydrolase, putative
**30**	**AFLA_090590**	-	alpha-1,2-mannosidase, putative subfamily
**30**	**AFLA_090600**	-	conserved hypothetical protein
**30**	**AFLA_090610**	-	hypothetical protein
**30**	**AFLA_090620**	-	hypothetical protein
**30**	**AFLA_090630**	-	cytochrome P450, putative
**30**	**AFLA_090640**	-	geranyl geranyl pyrophosphate synthase, putative
**30**	**AFLA_090660**	-	conserved hypothetical protein
**30**	**AFLA_090650**	-1.23	DUF1115 domain protein
**30**	**AFLA_090670**	1.11	hypothetical protein
**30**	**AFLA_090680**	1.92	cytochrome P450, putative
**30**	**AFLA_090690**	2.38	mycelial catalase Cat1
**31**	**AFLA_096340**	-	hypothetical protein
**31**	**AFLA_096350**	-	TrkA-N domain dehydrogenase, putative

**31**	**AFLA_096390**	-	prenyl transferase AtmC
**31**	**AFLA_096400**	-	FAD-dependent monooxygenase AtmM
**31**	**AFLA_096430**	-	cytochrome P450, putative
**31**	**AFLA_096410**	-3.01	PTH11-like integral membrane protein, putative
**31**	**AFLA_096330**	-2.40	C6 and C2H2 transcription factor
**31**	**AFLA_096420**	-1.66	hypothetical protein
**31**	**AFLA_096370**	2.41	Zn2Cys6 transcription factor
**31**	**AFLA_096360**	3.83	hypothetical protein
**31**	**AFLA_096380**	4.42	FAD dependent oxidoreductase, putative
**32**	**AFLA_096700**	-	NRPS-like enzyme, putative
**32**	**AFLA_096710**	-	NRPS-like enzyme, putative
**32**	**AFLA_096720**	-	hypothetical protein
**32**	**AFLA_096730**	-	conserved hypothetical protein
**32**	**AFLA_096770**	-	polyketide synthase, putative
**32**	**AFLA_096740**	-8.85	monoxygenase, putative
**32**	**AFLA_096760**	-7.35	monocarboxylate transporter, putative
**32**	**AFLA_096750**	-5.37	cytochrome P450, putative
**33**	**AFLA_017840**	-	NRPS-like enzyme, putative
**33**	**AFLA_017850**	-4.85	conserved hypothetical protein
**33**	**AFLA_017860**	-2.98	hypothetical protein
**34**	**AFLA_018250**	-	conserved hypothetical protein
**34**	**AFLA_018270**	-	hypothetical protein
**34**	**AFLA_018280**	-	mitochondrial outer membrane protein (Sam35), putative
**34**	**AFLA_018290**	-	conserved hypothetical protein
**34**	**AFLA_018300**	-	cell division control protein 14
**34**	**AFLA_018310**	-	geranylgeranyl diphosphate synthase
**34**	**AFLA_018320**	-	Smr domain protein
**34**	**AFLA_018340**	-	G-protein complex alpha subunit GpaA/FadA
**34**	**AFLA_018260**	-1.42	DUF543 domain protein
**34**	**AFLA_018350**	1.72	conserved hypothetical protein
**34**	**AFLA_018330**	3.00	hypothetical protein
**35**	**AFLA_038600**	-1.10	nonribosomal peptide synthase, putative
**35**	**AFLA_038590**	3.93	Ankyrin repeat protein
**35**	**AFLA_038580**	4.10	RTA1 domain protein
**35**	**AFLA_038570**	4.44	malonyl CoA-acyl carrier protein transacylase, putative
**36**	**AFLA_039200**	-	cytochrome P450, putative
**36**	**AFLA_039210**	-	cytochrome P450, putative
**36**	**AFLA_039220**	-	cytochrome P450, putative
**36**	**AFLA_039230**	-	short-chain dehydrogenase, putative

**36**	**AFLA_039240**	-	aristolochene synthase, putative
**36**	**AFLA_039270**	-	carboxylesterase, putative
**36**	**AFLA_039260**	2.02	esterase, putative
**36**	**AFLA_039250**	2.04	hypothetical protein
**37**	**AFLA_041590**	-	peroxisomal multifunctional beta-oxidation protein (MFP), putative
**37**	**AFLA_041600**	-	RING finger protein (Zin), putative
**37**	**AFLA_041610**	-	NRPS-like enzyme, putative
**37**	**AFLA_041620**	3.61	aquaporin, putative
**38**	**AFLA_042330**	-	conserved hypothetical protein
**38**	**AFLA_042340**	-	conserved hypothetical protein
**38**	**AFLA_042350**	-	conserved hypothetical protein
**38**	**AFLA_042360**	-	hypothetical protein
**38**	**AFLA_042370**	-	conserved hypothetical protein
**39**	**AFLA_045490**	-	dimethylallyl tryptophan synthase, putative
**39**	**AFLA_045510**	-	integral membrane protein
**39**	**AFLA_045500**	4.36	cytochrome P450, putative
**40**	**AFLA_100320**	-	hypothetical protein
**40**	**AFLA_100330**	-	FAD dependent oxidoreductase, putative
**40**	**AFLA_100340**	-	nonribosomal peptide synthase, putative
**40**	**AFLA_100360**	-	amino acid transporter, putative
**40**	**AFLA_100370**	-	acetylornithine aminotransferase, putative
**40**	**AFLA_100380**	-	hypothetical protein
**40**	**AFLA_100390**	-	conserved hypothetical protein
**40**	**AFLA_100400**	-	hypothetical protein
**40**	**AFLA_100410**	-	hypothetical protein
**40**	**AFLA_100420**	-	amidase, putative
**40**	**AFLA_100270**	-2.06	aliphatic nitrilase, putative
**40**	**AFLA_100280**	-1.96	MFS transporter, putative
**40**	**AFLA_100310**	-1.94	oxidoreductase, putative
**40**	**AFLA_100300**	-1.44	C6 transcription factor, putative
**40**	**AFLA_100290**	-1.43	aliphatic nitrilase, putative
**40**	**AFLA_100350**	3.46	conserved hypothetical protein
**41**	**AFLA_101740**	-6.59	glycerophosphoinositol/ glycerophosphocholine transporter, putative
**41**	**AFLA_101750**	-5.66	molybdopterin synthase small subunit CnxG
**41**	**AFLA_101760**	-4.09	molybdenum cofactor biosynthesis protein Gephyrin, putative
**41**	**AFLA_101720**	1.27	cytochrome P450, putative
**41**	**AFLA_101730**	2.08	ferulate-5-hydroxylase, putative

**41**	**AFLA_101700**	2.24	NRPS-like enzyme, putative
**41**	**AFLA_101710**	2.37	NmrA-like family protein
**41**	**AFLA_101690**	3.10	alcohol dehydrogenase, putative
**42**	**AFLA_102110**	-	conserved hypothetical protein
**42**	**AFLA_102150**	-	SAM dependent methyltransferase, putative
**42**	**AFLA_102140**	1.24	conserved hypothetical protein
**42**	**AFLA_102120**	1.25	hypothetical protein
**42**	**AFLA_102160**	2.89	lanosterol synthase, putative
**42**	**AFLA_102130**	3.34	conserved hypothetical protein
**43**	**AFLA_102480**	-	isoamyl alcohol oxidase
**43**	**AFLA_102490**	-	conserved hypothetical protein
**43**	**AFLA_102500**	-	9-cis-epoxycarotenoid dioxygenase, putative
**43**	**AFLA_102520**	-	phytoene dehydrogenase, putative
**43**	**AFLA_102530**	-	UMTA methyltransferase family protein
**43**	**AFLA_102540**	-	hypothetical protein
**43**	**AFLA_102550**	-	G-patch domain protein, putative
**43**	**AFLA_102510**	2.71	phytoene synthase, putative
**44**	**AFLA_064250**	-	phenol 2-monooxygenase, putative
**44**	**AFLA_064270**	-	gibberellin 2-oxidase, putative
**44**	**AFLA_064280**	-	N-methyltransferase, putative
**44**	**AFLA_064290**	-	O-methyltransferase, putative
**44**	**AFLA_064300**	-	fructosyl amino acid oxidase, putative
**44**	**AFLA_064330**	-	conserved hypothetical protein
**44**	**AFLA_064340**	-	conserved hypothetical protein
**44**	**AFLA_064260**	-1.01	oligopeptide transporter, putative
**44**	**AFLA_064320**	1.16	oligopeptide transporter, putative
**44**	**AFLA_064310**	1.21	hypothetical protein
**44**	**AFLA_064240**	1.54	nonribosomal peptide synthase, putative
**45**	**AFLA_064510**	-	thioredoxin reductase GliT-like, putative
**45**	**AFLA_064520**	-	O-methyltransferase GliM-like, putative
**45**	**AFLA_064610**	-	short-chain oxidoreductase, putative
**45**	**AFLA_064630**	-	hypothetical protein
**45**	**AFLA_064560**	-1.39	nonribosomal peptide synthase GliP-like, putative
**45**	**AFLA_064620**	-1.00	conserved hypothetical protein
**45**	**AFLA_064500**	1.33	conserved hypothetical protein
**45**	**AFLA_064580**	2.12	oxidoreductase, putative
**45**	**AFLA_064570**	2.17	uracil permease, putative
**45**	**AFLA_064540**	2.31	cytochrome P450 oxidoreductase GliC
**45**	**AFLA_064600**	2.34	conserved hypothetical protein

**45**	**AFLA_064550**	2.53	membrane dipeptidase GliJ-like, putative
**45**	**AFLA_064530**	2.63	glutathione S-transferase GliG-like, putative
**45**	**AFLA_064460**	2.88	conserved hypothetical protein
**45**	**AFLA_064440**	3.02	transport protein, putative
**45**	**AFLA_064590**	3.17	O-methyltransferase, putative
**45**	**AFLA_064480**	3.28	conserved hypothetical protein
**45**	**AFLA_064470**	3.31	cytochrome P450, putative
**45**	**AFLA_064450**	3.74	aminotransferase GliI-like, putative
**45**	**AFLA_064490**	3.82	conserved hypothetical protein
**45**	**AFLA_064640**	4.45	oxidoreductase, short-chain dehydrogenase/reductase family
**46**	**AFLA_066730**	-2.68	alcohol dehydrogenase, putative
**46**	**AFLA_066710**	1.68	conserved hypothetical protein
**46**	**AFLA_066700**	2.27	P450 family sporulation-specific N-formyltyrosine oxidase Dit2
**46**	**AFLA_066720**	2.34	nonribosomal peptide synthase, putative
**47**	**AFLA_066840**	-	hybrid PKS/NRPS enzyme, putative
**47**	**AFLA_066850**	-	conserved hypothetical protein
**47**	**AFLA_066860**	-	conserved hypothetical protein
**47**	**AFLA_066870**	-	conserved hypothetical protein
**47**	**AFLA_066900**	-1.60	conserved hypothetical protein
**47**	**AFLA_066970**	1.28	conserved hypothetical protein
**47**	**AFLA_066890**	1.36	cytochrome P450, putative
**47**	**AFLA_066950**	2.04	conserved hypothetical protein
**47**	**AFLA_066880**	2.08	monocarboxylate transporter, putative
**47**	**AFLA_066920**	2.36	alcohol dehydrogenase, putative
**47**	**AFLA_066910**	2.48	conserved hypothetical protein
**47**	**AFLA_066960**	3.82	hypothetical protein
**47**	**AFLA_066930**	3.86	cytochrome P450, putative
**47**	**AFLA_066940**	4.39	O-methyltransferase, putative
**48**	**AFLA_069340**	-2.33	MSF drug transporter, putative
**48**	**AFLA_069350**	-1.42	conserved hypothetical protein
**48**	**AFLA_069330**	1.19	nonribosomal peptide synthase Pes1
**49**	**AFLA_070860**	-	ACV synthetase PcbAB
**49**	**AFLA_070900**	-	conserved hypothetical protein
**49**	**AFLA_070920**	-	NRPS-like enzyme, putative
**49**	**AFLA_070890**	-3.79	hypothetical protein
**49**	**AFLA_070870**	1.52	isopenicillin N synthetase PcbC
**49**	**AFLA_070910**	1.79	aminotransferase, putative

**49**	**AFLA_070880**	3.90	acyl-coenzyme A:Isopenicillin N acyltransferase PenDE
**50**	**AFLA_002910**	-	conserved hypothetical protein
**50**	**AFLA_002930**	-	hypothetical protein
**50**	**AFLA_002920**	-4.66	flavonoid 3-hydroxylase, putative
**50**	**AFLA_002890**	-1.06	AMP-binding enzyme family protein
**50**	**AFLA_002900**	2.44	polyketide synthase, putative
**51**	**AFLA_004280**	-	C6 transcription factor, putative
**51**	**AFLA_004290**	-	conserved hypothetical protein
**51**	**AFLA_004300**	-5.05	prenyltransferase, putative
**52**	**AFLA_004440**	-10.38	ABC multidrug transporter, putative
**52**	**AFLA_004430**	-7.35	conserved hypothetical protein
**52**	**AFLA_004450**	-6.59	nonribosomal peptide synthase, putative
**53**	**AFLA_005270**	-	conserved hypothetical protein
**53**	**AFLA_005280**	-	hypothetical protein
**53**	**AFLA_005300**	-	ankyrin repeat-containing protein, putative
**53**	**AFLA_005320**	-	polyketide synthase, putative
**53**	**AFLA_005330**	-	conserved hypothetical protein
**53**	**AFLA_005340**	-	conserved hypothetical protein
**53**	**AFLA_005350**	-	hypothetical protein
**53**	**AFLA_005360**	-	conserved hypothetical protein
**53**	**AFLA_005380**	-	hypothetical protein
**53**	**AFLA_005400**	-	ankyrin repeat-containing protein, putative
**53**	**AFLA_005390**	-3.83	conserved hypothetical protein
**53**	**AFLA_005290**	-1.14	conserved hypothetical protein
**53**	**AFLA_005410**	-1.05	5-AMP-activated protein kinase, putative
**53**	**AFLA_005310**	5.04	vacuolar ATP synthase proteolipid subunit, putative
**53**	**AFLA_005370**	5.80	conserved hypothetical protein
**54**	**AFLA_005440**	-1.70	nonribosomal peptide synthase, putative
**54**	**AFLA_005450**	1.33	conserved hypothetical protein
**55**	**AFLA_006110**	-	conserved hypothetical protein
**55**	**AFLA_006130**	-	NADH pyrophosphatase, putative
**55**	**AFLA_006140**	-	conserved hypothetical protein
**55**	**AFLA_006150**	-	flavin dependent monooxygenase, putative
**55**	**AFLA_006160**	-	HDA1 complex subunit, putative
**55**	**AFLA_006180**	-	conidial pigment biosynthesis oxidase Arb2/brown2
**55**	**AFLA_006190**	-	multicopper oxidase, putative
**55**	**AFLA_006240**	-	PHD transcription factor (Rum1), putative
**55**	**AFLA_006170**	-5.70	polyketide synthetase PksP
**55**	**AFLA_006100**	-2.45	HypA-like protein, putative

**55**	**AFLA_006230**	-1.88	1-aminocyclopropane-1-carboxylate deaminase, putative
**55**	**AFLA_006120**	-1.26	glutamine dependent NAD+ synthetase, putative
**55**	**AFLA_006220**	-1.22	exopolyphosphatase, putative
**55**	**AFLA_006210**	1.43	SH3 domain protein (Cyk3), putative
**55**	**AFLA_006200**	3.15	hypothetical protein
**56**	**AFLA_006800**	-	DNA damage repair protein (Rad9), putative
**56**	**AFLA_006810**	-	50S ribosomal protein L27, putative
**56**	**AFLA_006830**	-	scramblase family protein
**56**	**AFLA_006860**	-	oxidosqualene:lanosterol cyclase
**56**	**AFLA_006870**	-	SGT1 and CS domain protein
**56**	**AFLA_006900**	-	cyclin-dependent protein kinase PhoA
**56**	**AFLA_006920**	-3.09	cAMP receptor-like protein, putative
**56**	**AFLA_006880**	-2.68	PQ loop repeat protein
**56**	**AFLA_006890**	-1.82	SIR2 family histone deacetylase, putative
**56**	**AFLA_006840**	-1.39	ubiquitin conjugating enzyme (UbcH), putative
**56**	**AFLA_006850**	1.02	meiosis-specific topoisomerase Spo11, putative
**56**	**AFLA_006820**	1.64	conserved hypothetical protein
**56**	**AFLA_006910**	2.74	hypothetical protein
**57**	**AFLA_135430**	-	cytochrome P450, putative
**57**	**AFLA_135440**	-	cytochrome P450, putative
**57**	**AFLA_135450**	-	trichodiene synthase, putative
**57**	**AFLA_135470**	-	ornithine decarboxylase, putative
**57**	**AFLA_135480**	-	galactose-proton symport, putative
**57**	**AFLA_135490**	-	nonribosomal peptide synthase, putative
**57**	**AFLA_135460**	-1.66	conserved hypothetical protein
**58**	**AFLA_137790**	-	cell wall protein, putative
**58**	**AFLA_137800**	-	conserved hypothetical protein
**58**	**AFLA_137810**	-	pantothenate transporter, putative
**58**	**AFLA_137890**	-	conserved hypothetical protein
**58**	**AFLA_137880**	1.22	extracellular triacylglycerol lipase, putative
**58**	**AFLA_137830**	1.77	hypothetical protein
**58**	**AFLA_137780**	2.50	ABC transporter, putative
**58**	**AFLA_137820**	2.92	conserved hypothetical protein
**58**	**AFLA_137840**	4.15	integral membrane protein
**58**	**AFLA_137870**	5.11	polyketide synthase, putative
**58**	**AFLA_137850**	5.50	conserved hypothetical protein
**58**	**AFLA_137860**	5.64	conserved hypothetical protein
**59**	**AFLA_139160**	-	aflX/ ordB/ monooxygenase/ oxidase
**59**	**AFLA_139180**	-	aflV/ cypX/ cytochrome P450 monooxygenase

**59**	**AFLA_139190**	-	aflK/ vbs/ VERB synthase
**59**	**AFLA_139200**	-	aflQ/ ordA/ ord-1/ oxidoreductase/ cytochrome P450 monooxigenase
**59**	**AFLA_139210**	-	aflP/ omtA/ omt-1/ O-methyltransferase A
**59**	**AFLA_139220**	-	aflO/ omtB/ dmtA/ O-methyltransferase B
**59**	**AFLA_139230**	-	aflI/ avfA/ cytochrome P450 monooxygenase
**59**	**AFLA_139250**	-	aflL/ verB/ desaturase/ P450 monooxygenase
**59**	**AFLA_139260**	-	aflG/ avnA/ ord-1/ cytochrome P450 monooxygenase
**59**	**AFLA_139280**	-	aflN/ verA/ monooxygenase
**59**	**AFLA_139300**	-	aflM/ ver-1/ dehydrogenase/ ketoreductase
**59**	**AFLA_139310**	-	aflE/ norA/ aad/ adh-2/ NOR reductase/ dehydrogenase
**59**	**AFLA_139320**	-	aflJ/ estA/ esterase
**59**	**AFLA_139330**	-	aflH/ adhA/ short chain alcohol dehydrogenase
**59**	**AFLA_139390**	-	aflD / nor-1 / reductase
**59**	**AFLA_139400**	-	aflCa / hypC / hypothetical protein
**59**	**AFLA_139440**	-	aflF / norB / dehydrogenase
**59**	**AFLA_139340**	-3.16	aflS/ pathway regulator
**59**	**AFLA_139360**	-2.99	aflR / apa-2 / afl-2 / transcription activator
**59**	**AFLA_139380**	-2.57	aflA / fas-2 / hexA / fatty acid synthase alpha subunit
**59**	**AFLA_139150**	1.16	aflY/ hypA/ hypP/ hypothetical protein
**59**	**AFLA_139370**	1.47	aflB / fas-1 / fatty acid synthase beta subunit
**59**	**AFLA_139410**	2.00	aflC / pksA / pksL1 / polyketide synthase
**59**	**AFLA_139170**	2.05	aflW/ moxY/ monooxygenase
**59**	**AFLA_139420**	2.73	aflT / aflT / transmembrane protein
**59**	**AFLA_139430**	4.24	aflU / cypA / P450 monooxygenase
**60**	**AFLA_139590**	-	choline dehydrogenase, putative
**60**	**AFLA_139600**	-	glucose-methanol-choline (gmc) oxidoreductase, putative
**60**	**AFLA_139620**	-	amino acid permease family protein
**60**	**AFLA_139630**	-	ABC multidrug transporter, putative
**60**	**AFLA_139640**	-	hypothetical protein
**60**	**AFLA_139650**	-	hypothetical protein
**60**	**AFLA_139660**	-	hypothetical protein
**60**	**AFLA_139670**	-	nonribosomal peptide synthase, putative
**60**	**AFLA_139610**	1.75	ceramidase, putative
**61**	**AFLA_022840**	-	allantoate permease, putative
**61**	**AFLA_022850**	-	conserved hypothetical protein
**61**	**AFLA_022870**	-	conserved hypothetical protein
**61**	**AFLA_023000**	-	Ankyrin domain protein
**61**	**AFLA_023010**	-	GA4 desaturase family protein

**61**	**AFLA_023030**	-	cytochrome P450 oxidoreductase GliC-like, putative
**61**	**AFLA_023040**	-	C6 transcription factor, putative
**61**	**AFLA_023060**	-	hypothetical protein
**61**	**AFLA_023080**	-	integral membrane protein TmpA
**61**	**AFLA_023070**	-2.93	integral membrane protein
**61**	**AFLA_022990**	-1.83	conserved hypothetical protein
**61**	**AFLA_022880**	-1.54	oligopeptide transporter, putative
**61**	**AFLA_022860**	1.73	hypothetical protein
**61**	**AFLA_023050**	1.99	MFS transporter, putative
**61**	**AFLA_023020**	4.15	NRPS-like enzyme, putative
**62**	**AFLA_027210**	-	conserved hypothetical protein
**62**	**AFLA_027220**	-	hypothetical protein
**62**	**AFLA_027230**	-	alpha-aminoadipate reductase Lys2, putative
**62**	**AFLA_027250**	-	aldo-keto reductase, putative
**62**	**AFLA_027260**	-	conserved hypothetical protein
**62**	**AFLA_027200**	1.28	xanthine dehydrogenase HxA, putative
**62**	**AFLA_027240**	1.58	carbonic anhydrase Nce103, putative
**63**	**AFLA_028730**	-	conserved hypothetical protein
**63**	**AFLA_028740**	-	coenzyme A transferase, putative
**63**	**AFLA_028760**	-1.30	C2H2 transcription factor, putative
**63**	**AFLA_028720**	-1.20	NRPS-like enzyme, putative
**63**	**AFLA_028710**	2.28	short chain type dehydrogenase, putative
**63**	**AFLA_028750**	2.49	UMTA methyltransferase family protein
**64**	**AFLA_105000**	-	DUF636 domain protein
**64**	**AFLA_105040**	-	conserved hypothetical protein
**64**	**AFLA_105060**	-	NAD dependent epimerase/dehydratase, putative
**64**	**AFLA_105080**	-	terpene synthase family protein
**64**	**AFLA_105030**	-2.57	cytosine deaminase, putative
**64**	**AFLA_105010**	-2.53	methyltransferase, putative
**64**	**AFLA_105020**	-1.54	conserved hypothetical protein
**64**	**AFLA_105050**	-1.45	geranylgeranyl pyrophosphate synthetase AtmG, putative
**64**	**AFLA_105070**	-1.13	cytochrome P450 oxygenase, putative
**65**	**AFLA_105130**	-	conserved hypothetical protein
**65**	**AFLA_105140**	-	conserved hypothetical protein
**65**	**AFLA_105150**	-	extracellular carboxylesterase, putative
**65**	**AFLA_105160**	-	hypothetical protein
**65**	**AFLA_105190**	-4.46	NRPS-like enzyme, putative
**65**	**AFLA_105180**	-2.54	hypothetical protein
**65**	**AFLA_105170**	-2.39	O-methyltransferase, putative

**65**	**AFLA_105120**	-1.50	GNAT family acetyltransferase, putative
**66**	**AFLA_105370**	-	conserved hypothetical protein
**66**	**AFLA_105380**	-	actin-binding protein, putative
**66**	**AFLA_105400**	-	kinesin family protein (KlpA), putative
**66**	**AFLA_105440**	-	cytochrome P450, putative
**66**	**AFLA_105450**	-	polyketide synthase, putative
**66**	**AFLA_105460**	-	conserved hypothetical protein
**66**	**AFLA_105490**	-	phosphoribulokinase/uridine kinase family protein
**66**	**AFLA_105480**	-5.35	DUF636 domain protein
**66**	**AFLA_105470**	-3.91	translation initiation factor eIF-2B subunit family protein
**66**	**AFLA_105540**	-2.34	MFS multidrug transporter, putative
**66**	**AFLA_105530**	-2.27	C6 transcription factor, putative
**66**	**AFLA_105390**	-1.20	conserved hypothetical protein
**66**	**AFLA_105520**	1.07	conserved hypothetical protein
**66**	**AFLA_105510**	1.33	conserved hypothetical protein
**66**	**AFLA_105430**	1.37	fatty acid desaturase protein, putative
**66**	**AFLA_105410**	1.97	thioester reductase family protein, putative
**66**	**AFLA_105420**	2.16	hypothetical protein
**66**	**AFLA_105500**	4.17	conserved hypothetical protein
**67**	**AFLA_107100**	-	conserved hypothetical protein
**67**	**AFLA_107120**	-	conserved hypothetical protein
**67**	**AFLA_107130**	-	D-isomer specific 2-hydroxyacid dehydrogenase family protein
**67**	**AFLA_107140**	-	conserved hypothetical protein
**67**	**AFLA_107110**	-1.93	conserved hypothetical protein
**67**	**AFLA_107150**	1.94	conserved hypothetical protein
**68**	**AFLA_108540**	-	NADH oxidase, putative
**68**	**AFLA_108550**	-	polyketide synthase, putative
**68**	**AFLA_108560**	-	O-methyltransferase, putative
**68**	**AFLA_108570**	-	conserved hypothetical protein
**68**	**AFLA_108580**	-	cytochrome P450, putative
**69**	**AFLA_109390**	-	conserved hypothetical protein
**69**	**AFLA_109400**	-	extracellular protein, putative
**69**	**AFLA_109410**	-	UV-endonuclease UVE-1
**69**	**AFLA_109420**	-	actin cytoskeleton organization protein App1, putative
**69**	**AFLA_109440**	-	long-chain-fatty-acid-CoA ligase, putative
**69**	**AFLA_109380**	-2.84	alcohol dehydrogenase, putative
**69**	**AFLA_109430**	4.23	nonribosomal siderophore peptide synthase SidC
**70**	**AFLA_112780**	-	conserved hypothetical protein

**70**	**AFLA_112790**	-	acyl-CoA thioester hydrolase, putative
**70**	**AFLA_112810**	-	trytophan synthase alpha subunit, putative
**70**	**AFLA_112820**	-	toxin biosynthesis ketoreductase, putative
**70**	**AFLA_112830**	-	conserved hypothetical protein
**70**	**AFLA_112840**	-	polyketide synthase, putative
**70**	**AFLA_112880**	-	2,4-dichlorophenol 6-monooxygenase, putative
**70**	**AFLA_112900**	-	epoxide hydrolase, putative
**70**	**AFLA_112910**	-	conserved hypothetical protein
**70**	**AFLA_112920**	-	mitochondrial cytochrome b2-like, putative
**70**	**AFLA_112800**	-4.33	L-lactate dehydrogenase
**70**	**AFLA_112870**	1.11	monooxygenase, putative
**70**	**AFLA_112890**	1.30	MFS transporter, putative
**70**	**AFLA_112860**	1.82	conserved hypothetical protein
**70**	**AFLA_112850**	3.07	O-methyltransferase, putative
**71**	**AFLA_059980**	3.75	choline dehydrogenase, putative
**71**	**AFLA_060020**	4.04	PKS-like enzyme, putative
**71**	**AFLA_059960**	4.33	conserved hypothetical protein
**71**	**AFLA_059970**	4.65	short-chain dehydrogenase, putative
**71**	**AFLA_059990**	4.88	O-methyltransferase, putative
**71**	**AFLA_060000**	5.07	metallo-beta-lactamase domain protein
**71**	**AFLA_060010**	5.88	PKS-like enzyme, putative
**71**	**AFLA_059950**	7.37	oxidoreductase, FAD-binding, putative
**72**	**AFLA_060650**	-	glutaminyl-tRNA synthetase
**72**	**AFLA_060660**	-	AMP-binding enzyme, putative
**72**	**AFLA_060670**	-	integral membrane protein
**72**	**AFLA_060680**	1.60	conserved hypothetical protein
**73**	**AFLA_062440**	-	conserved hypothetical protein
**73**	**AFLA_062450**	-	sphingomyelin phosphodiesterase, putative
**73**	**AFLA_062460**	-	non-classical export protein Nce102, putative
**73**	**AFLA_062470**	-	pentalenene synthase, putative
**73**	**AFLA_062480**	-	conserved hypothetical protein
**73**	**AFLA_062500**	-	mitogen-activated protein kinase MAF1
**73**	**AFLA_062510**	-	myosin heavy chain, embryonic smooth muscle isoform, putative
**73**	**AFLA_062520**	-	COPII-coated vesicle protein (Erv41), putative
**73**	**AFLA_062430**	-5.13	non-classical export protein Nce102, putative
**73**	**AFLA_062490**	1.25	COPII-coated vesicle protein (Erv41), putative
**74**	**AFLA_062830**	-	monoxygenase, putative
**74**	**AFLA_062870**	-	conserved hypothetical protein

**74**	**AFLA_062880**	-	short chain dehydrogenase/oxidoreductase, putative
**74**	**AFLA_062890**	-	hypothetical protein
**74**	**AFLA_062900**	-	glycerol-3-phosphate O-acyltransferase, putative
**74**	**AFLA_062910**	-	proline-specific permease, putative
**74**	**AFLA_062920**	-	conserved hypothetical protein
**74**	**AFLA_062930**	-	glycosyl hydrolase, family 43, putative
**74**	**AFLA_062840**	-3.46	short chain dehydrogenase/oxidoreductase, putative
**74**	**AFLA_062860**	-1.15	glycerol-3-phosphate O-acyltransferase, putative
**74**	**AFLA_062810**	1.15	fatty acid desaturase, putative
**74**	**AFLA_062850**	3.26	hypothetical protein
**74**	**AFLA_062820**	3.38	polyketide synthase, putative
**74**	**AFLA_062800**	3.98	conserved hypothetical protein
