# Supplementary material for: WetA bridges cellular and chemical development in Aspergillus flavus
Source: PLoS One. 2017 Jun 28;12(6):e0179571. doi: 10.1371/journal.pone.0179571 (PMC5489174; doi:10.1371/journal.pone.0179571)
Supplement: S9 Table — (PDF) [file pone.0179571.s011.pdf]

S9 Table. Brief description of WetA-regulated genes associated with conidiation

Genes	*Function*	*References*
brlA	Initiation of conidiation	[1]
abaA	Phialides formation	[1]
stuA	Required for conidiophore formation and morphology	[1]
medA	Required for conidiophore formation and morphology	[1]
veA	Distinct roles in conidiation	[2]
vosA	Repressor of conidiation	[3]
steC	Component of MAP pathway coordinating sexual and asexual development	[4]
vapA	Positive regulator of conidiation	[5]
vipC	Positive regulator of conidiation	[5]
fphA	Red-light-dependent positive regulator of conidiation	[6]
llmF	Light-dependent positive regulator of conidiation	[7]
ppoA	Negative regulator of conidiation	[8]
ppoB	Negative regulator of conidiation	[8]
ppoC	Positive regulator of conidiation	[8]
nsdD	Repressor of conidiation	[9]
flbC	Required for the activation of *brlA*	[10]
fluG	Positive regulator of conidiation	[11]
sfgA	Negative regulator of conidiation	[12]
pkaA	Negative regulator of conidiation	[13]
flbA	Attenuates GpaA-mediated signaling	[10]
ricA	Putative activator of G protein signaling	[14]
sltA	Positive regulator of conidiation	[15]
rgdA	Positive regulator of conidiation	[16]
mtfA	Positive regulator of conidiation	[17]
rhbA	Negative regulator of conidiation	[18]
nsdC	Negative regulator of conidiation	[19]
osaA	Negative regulator of conidiation	[20]

1. Clutterbuck AJ. A mutational analysis of conidial development in *Aspergillus nidulans*. Genetics. 1969;63: 317–327. Available: http://www.genetics.org/content/63/2/317.short

2. Clutterbuck AJ. The Genetics of Conidiation in *Aspergillus nidulans*. In: SmithJ, PatemanJ, editors. Genetics and Physiology of Aspergillus. London: Academic Press; 1977. pp. 305–317.

3. Ni M, Yu J-H. A novel regulator couples sporogenesis and trehalose biogenesis in *Aspergillus nidulans*. PLoS One. Public Library of Science; 2007;2: e970. doi:10.1371/journal.pone.0000970

4. Wei H, Requena N, Fischer R. The MAPKK kinase SteC regulates conidiophore morphology and is essential for heterokaryon formation and sexual development in the homothallic fungus *Aspergillus nidulans*. Mol Microbiol. 2003;47: 1577–88. Available: http://www.ncbi.nlm.nih.gov/pubmed/12622813

5. Sarikaya-Bayram Ö, Bayram Ö, Feussner K, Kim J-H, Kim H-S, Kaever A, et al. Membrane-bound methyltransferase complex VapA-VipC-VapB guides epigenetic control of fungal development. Dev Cell. 2014;29: 406–420. doi:10.1016/j.devcel.2014.03.020

6. Hatakeyama R, Nakahama T, Higuchi Y, Kitamoto K. Light represses conidiation in koji mold *Aspergillus oryzae*. Biosci Biotechnol Biochem. Japan Society for Bioscience, Biotechnology, and Agrochemistry; 2007;71: 1844–1849. doi:10.1271/bbb.60713

7. Palmer JM, Theisen JM, Duran RM, Grayburn WS, Calvo AM, Keller NP. Secondary metabolism and development is mediated by LlmF control of VeA subcellular localization in *Aspergillus nidulans*. Heitman J, editor. PLoS Genet. Public Library of Science; 2013;9: e1003193. doi:10.1371/journal.pgen.1003193

8. Tsitsigiannis DI, Kowieski TM, Zarnowski R, Keller NP. Three putative oxylipin biosynthetic genes integrate sexual and asexual development in *Aspergillus nidulans*. Microbiology. 2005;151: 1809–1821. doi:10.1099/mic.0.27880-0

9. Lee M-K, Kwon N-J, Choi JM, Lee I-S, Jung S, Yu J-H. NsdD is a key repressor of asexual development in *Aspergillus nidulans*. Genetics. 2014;197: 159–173. doi:10.1534/genetics.114.161430

10. Wieser J, Lee BN, Fondon J, Adams TH. Genetic requirements for initiating asexual development in *Aspergillus nidulans*. Curr Genet. 1994;27: 62–9. Available: http://www.ncbi.nlm.nih.gov/pubmed/7750148

11. Yager LN. Early developmental events during asexual and sexual sporulation in *Aspergillus nidulans*. Biotechnology. 1992;23: 19–41. Available: http://www.ncbi.nlm.nih.gov/pubmed/1504597

12. Seo J-A, Guan Y, Yu J-H. Suppressor mutations bypass the requirement of *fluG* for asexual sporulation and sterigmatocystin production in *Aspergillus nidulans*. Genetics. 2003;165: 1083–93. Available: http://www.ncbi.nlm.nih.gov/pubmed/14668366

13. Shimizu K, Keller NP. Genetic Involvement of a cAMP-dependent protein kinase in a G protein signaling pathway regulating morphological and chemical transitions in *Aspergillus nidulans*. Genetics. 2001;157: 591–600. Available: http://www.genetics.org/content/157/2/591.abstract

14. Kwon N-J, Park H-S, Jung S, Kim SC, Yu J-H. The putative guanine nucleotide exchange factor RicA mediates upstream signaling for growth and development in *Aspergillus*. Eukaryot Cell. 2012;11: 1399–1412. doi:10.1128/EC.00255-12

15. Shantappa S, Dhingra S, Hernández-Ortiz P, Espeso EA, Calvo AM. Role of the zinc finger

transcription factor *sltA* in morphogenesis and sterigmatocystin biosynthesis in the fungus *Aspergillus nidulans*. GoldmanGH, editor. PLoS One. 2013;8: e68492. doi:10.1371/journal.pone.0068492

16. Lee J-Y, Kim L-H, Kim H-E, Park J-S, Han K-H, Han D-M. A putative APSES transcription factor is necessary for normal growth and development of *Aspergillus nidulans*. J Microbiol. 2013;51: 800–6. doi:10.1007/s12275-013-3100-2

17. Ramamoorthy V, Dhingra S, Kincaid A, Shantappa S, Feng X, Calvo AM. The putative C_2H_2 transcription factor MtfA is a novel regulator of secondary metabolism and morphogenesis in *Aspergillus nidulans*. Goldman GH, editor. PLoS One. 2013;8: e74122. doi:10.1371/journal.pone.0074122

18. Harispe L, Portela C, Scazzocchio C, Penalva MA, Gorfinkiel L. Ras GTPase-Activating Protein Regulation of Actin Cytoskeleton and Hyphal Polarity in Aspergillus nidulans. Eukaryot Cell. 2008;7: 141–153. doi:10.1128/EC.00346-07

19. KimH-R, ChaeK-S, HanK-H, HanD-M. The *nsdC* gene encoding a putative C_2H_2-type transcription factor is a key activator of sexual development in *Aspergillus nidulans*. Genetics. 2009;182: 771–783. doi:10.1534/genetics.109.101667

20. Alkahyyat F, Ni M, Kim SC, Yu J-H. The WOPR domain protein OsaA orchestrates development in *Aspergillus nidulans*. Harris S, editor. PLoS One. 2015;10: e0137554. doi:10.1371/journal.pone.0137554
