# Supplementary material for: WetA bridges cellular and chemical development in Aspergillus flavus
Source: PLoS One. 2017 Jun 28;12(6):e0179571. doi: 10.1371/journal.pone.0179571 (PMC5489174; doi:10.1371/journal.pone.0179571)
Supplement: S10 Table — (PDF) [file pone.0179571.s012.pdf]

**S10 Table. DEGs predicted to encode transcription factors.**

| Gene ID | $Log_2$ Fold Change | Description | Type |
|---|---|---|---|
| **AFLA_122410** | 7.84 | Putative TF | bZIP |
| **AFLA_040300** | 7.03 | Putative TF | $C_6$ |
| **AFLA_094010** | 6.01 | *atf21* | bZIP |
| **AFLA_000010** | 4.85 | Putative TF | Zinc-binding |
| **AFLA_049410** | 4.68 | Putative TF | $C_6$ |
| **AFLA_085880** | 4.19 | Putative TF | BTB |
| **AFLA_103640** | 4.14 | *fcr1* | $C_6$ |
| **AFLA_079250** | 4.13 | Putative TF | Zinc-binding |
| **AFLA_120470** | 4.06 | *silA* ortholog | Zinc-binding |
| **AFLA_007370** | 3.81 | Putative TF | Zinc-binding |
| **AFLA_096100** | 3.73 | Putative TF | |
| **AFLA_139560** | 3.60 | Putative TF | $C_6$ |
| **AFLA_001310** | 3.57 | Putative TF | Zinc-binding |
| **AFLA_120480** | 3.15 | *silA* | Zinc-binding |
| **AFLA_120780** | 3.10 | Putative TF | $C_6$ |
| **AFLA_139360** | 2.99 | *aflR* | $C_6$ |
| **AFLA_051880** | 2.92 | *prnA* | $C_6$ |
| **AFLA_097720** | 2.91 | *clrA* | $C_6$ |
| **AFLA_123530** | 2.86 | Putative TF | Zinc-binding |
| **AFLA_096320** | 2.84 | Putative TF | C6 / C2H2 |
| **AFLA_005520** | 2.82 | *sfgA* | $C_6$ |
| **AFLA_126910** | 2.72 | Putative TF | $C_6$ |
| **AFLA_059110** | 2.72 | Putative TF | Zinc-binding |
| **AFLA_009580** | 2.63 | Putative TF | $C_6$ |
| **AFLA_065310** | 2.57 | Putative TF | Zinc-binding |
| **AFLA_124010** | 2.44 | Putative TF | $C_6$ |
| **AFLA_064980** | 2.44 | *sdrA* | $C_6$ |
| **AFLA_096330** | 2.40 | Putative TF | C6 / C2H2 |
| **AFLA_038210** | 2.39 | Putative TF | $C_6$ |
| **AFLA_122500** | 2.37 | Putative TF | $C_6$ |
| **AFLA_134920** | 2.36 | Putative TF | $C_6$ |
| **AFLA_009690** | 2.31 | Putative TF | Zinc-binding |
| **AFLA_034610** | 2.28 | Putative TF | $C_6$ |
| **AFLA_105530** | 2.27 | Putative TF | $C_6$ |
| **AFLA_033480** | 2.26 | Putative TF | $C_6$ |
| **AFLA_041330** | 2.22 | Putative TF | $C_6$ |

| | | | |
|---|---|---|---|
| **AFLA_123540** | 2.22 | Putative TF | Zinc-binding |
| **AFLA_136880** | 2.19 | Putative TF | $C_6$ |
| **AFLA_012100** | 2.16 | *pcaG* | NDT80_PhoG |
| **AFLA_093070** | 2.15 | Putative TF | $C_2H_2$ |
| **AFLA_049640** | 2.06 | Putative TF | Zinc-binding |
| **AFLA_119890** | 2.01 | Putative TF | $C_6$ |
| **AFLA_062330** | 1.93 | Putative TF | $C_6$ |
| **AFLA_076330** | 1.90 | Putative TF | Zinc-binding |
| **AFLA_124630** | 1.87 | Putative TF | Zinc-binding |
| **AFLA_000720** | 1.85 | Putative TF | Zinc-binding |
| **AFLA_123770** | 1.81 | *scfA* ortholog | $C_6$ |
| **AFLA_014270** | 1.81 | Putative TF | $C_6$ |
| **AFLA_080780** | 1.81 | Putative TF | Zinc-binding |
| **AFLA_120290** | 1.80 | Putative TF | $C_6$ |
| **AFLA_000730** | 1.79 | Putative TF | $C_6$ |
| **AFLA_075420** | 1.78 | Putative TF | Homeobox |
| **AFLA_079320** | 1.77 | Putative TF | Zinc-binding |
| **AFLA_138930** | 1.72 | Putative TF | $C_6$ |
| **AFLA_042930** | 1.71 | Putative TF | Zinc-binding |
| **AFLA_025720** | 1.69 | *nosA* | $C_6$ |
| **AFLA_025860** | 1.65 | Putative TF | $C_6$ |
| **AFLA_015850** | 1.62 | Putative TF | $C_6$ |
| **AFLA_125590** | 1.61 | Putative TF | Zinc-binding |
| **AFLA_118080** | 1.60 | *rdr1* | Zinc-binding |
| **AFLA_097920** | 1.59 | *scfA* | $C_6$ |
| **AFLA_053760** | 1.58 | Putative TF | Zinc-binding |
| **AFLA_110020** | 1.57 | Putative TF | $C_6$ |
| **AFLA_097740** | 1.49 | Putative TF | Zinc-binding |
| **AFLA_012010** | 1.49 | *ctf1B* | $C_6$ |
| **AFLA_030600** | 1.45 | *fkh1* | Forkhead |
| **AFLA_073870** | 1.45 | *regA* | $C_6$ |
| **AFLA_100300** | 1.44 | Putative TF | $C_6$ |
| **AFLA_068100** | 1.43 | Putative TF | Zinc-binding |
| **AFLA_049270** | 1.42 | *galX* | $C_6$ |
| **AFLA_036490** | 1.36 | *nscR* | $C_6$ |
| **AFLA_063720** | 1.33 | Putative TF | Zinc-binding |
| **AFLA_028760** | 1.30 | Putative TF | $C_2H_2$ |
| **AFLA_014120** | 1.30 | *scfA* ortholog | $C_6$ |
| **AFLA_048870** | 1.29 | *amdA* | $C_2H_2$ |

| | | | |
|---|---|---|---|
| **AFLA_042030** | 1.29 | Putative TF | $C_6$ |
| **AFLA_033160** | 1.27 | *sfp1* | $C_2H_2$ |
| **AFLA_097680** | 1.26 | Putative TF | $C_6$ |
| **AFLA_010880** | 1.26 | Putative TF | Zinc-binding |
| **AFLA_053230** | 1.26 | Putative TF | Zinc-binding |
| **AFLA_015920** | 1.25 | *aro80* | $C_6$ |
| **AFLA_139110** | 1.24 | *aflYd* | Zinc-binding |
| **AFLA_048920** | 1.24 | Putative TF | $C_6$ |
| **AFLA_123500** | 1.19 | Putative TF | $C_6$ |
| **AFLA_024580** | 1.19 | Putative TF | $C_6$ |
| **AFLA_018110** | 1.18 | Putative TF | bZIP |
| **AFLA_076320** | 1.18 | Putative TF | $C_6$ |
| **AFLA_043710** | 1.17 | Putative TF | $C_6$ |
| **AFLA_013890** | 1.17 | Putative TF | $C_2H_2$ |
| **AFLA_083510** | 1.14 | Putative TF | Homeobox/ $C_2H_2$ |
| **AFLA_090160** | 1.13 | Putative TF | Zinc-binding |
| **AFLA_010240** | 1.13 | Putative TF | Zinc-binding |
| **AFLA_121770** | 1.12 | Putative TF | Zinc-binding |
| **AFLA_098130** | 1.11 | Putative TF | Zinc-binding |
| **AFLA_064370** | 1.08 | Putative TF | $C_6$ |
| **AFLA_087810** | 1.07 | *metR* | bZIP |
| **AFLA_028560** | 1.06 | *amdR* | $C_6$ |
| **AFLA_017040** | 1.03 | Putative TF | $C_6$ |
| **AFLA_028410** | 1.02 | *pbcR* | $C_6$ |
| **AFLA_009490** | 1.01 | Putative TF | $C_6$ |
| **AFLA_118300** | -1.00 | Putative TF | $C_6$ |
| **AFLA_023420** | -1.01 | Putative TF | Zinc-binding |
| **AFLA_076040** | -1.04 | Putative TF | Zinc-binding |
| **AFLA_036190** | -1.10 | *rap1* | |
| **AFLA_089270** | -1.11 | *hacA* | bZIP |
| **AFLA_083820** | -1.13 | Putative TF | $C_6$ |
| **AFLA_048650** | -1.14 | *steA* | Homeobox/ Zinc-binding |
| **AFLA_030580** | -1.17 | *pacC* | $C_2H_2$ |
| **AFLA_080270** | -1.17 | Putative TF | $C_2H_2$ |
| **AFLA_097380** | -1.21 | Putative TF | CP2 |
| **AFLA_020130** | -1.21 | *rgdA* | APSES |
| **AFLA_018410** | -1.21 | Putative TF | $C_2H_2$ |
| **AFLA_129530** | -1.22 | Putative TF | Zinc-binding |

| | | | |
|---|---|---|---|
| **AFLA_074060** | -1.28 | Putative TF | NF-X1 |
| **AFLA_051900** | -1.35 | *cnjB* | Zinc-binding |
| **AFLA_067300** | -1.37 | Putative TF | Zinc-binding |
| **AFLA_074200** | -1.41 | Putative TF | $C_6$ |
| **AFLA_135110** | -1.44 | Putative TF | HLH |
| **AFLA_131640** | -1.47 | *devR/hpa3* | HLH |
| **AFLA_070980** | -1.48 | Putative TF | $C_6$ |
| **AFLA_038860** | -1.49 | Putative TF | Zinc-binding |
| **AFLA_058610** | -1.54 | *srbA* | HLH |
| **AFLA_104780** | -1.57 | Putative TF | $C_6$ |
| **AFLA_127920** | -1.57 | *crzA* | $C_2H_2$ |
| **AFLA_002290** | -1.58 | *amdX* | $C_2H_2$ |
| **AFLA_084200** | -1.59 | Putative TF | $C_6$ |
| **AFLA_050970** | -1.61 | Putative TF | Zinc-binding |
| **AFLA_020210** | -1.68 | *nsdD* | GATA |
| **AFLA_109220** | -1.73 | Putative TF | $C_6$ |
| **AFLA_050250** | -1.75 | *cpcA* | bZIP |
| **AFLA_099460** | -1.76 | *rfeG* | |
| **AFLA_083100** | -1.81 | *zipA* | bZIP |
| **AFLA_044680** | -1.88 | *ndtA* | NDT80_PhoG domain |
| **AFLA_088390** | -2.07 | *egd1* | btf3-like |
| **AFLA_035590** | -2.12 | Putative TF | $C_6$ |
| **AFLA_026250** | -2.16 | *rfeB* | Homeobox |
| **AFLA_071330** | -2.18 | Putative TF | Zinc-binding |
| **AFLA_110650** | -2.23 | *seb1* | $C_2H_2$ |
| AFLA_091490 | -2.27 | *mtfA* | $C_2H_2$ |
| **AFLA_046990** | -2.32 | *stuA* | APSES |
| **AFLA_064960** | -2.36 | Putative TF | Zinc-binding |
| **AFLA_096370** | -2.41 | Putative TF | $C_6$ |
| **AFLA_008120** | -2.47 | Putative TF | Zinc-binding |
| **AFLA_057080** | -2.48 | Putative TF | Zinc-binding |
| **AFLA_067290** | -2.60 | Putative TF | Zinc-binding |
| **AFLA_083560** | -2.61 | Putative TF | $C_6$ |
| **AFLA_119280** | -2.63 | Putative TF | Zinc-binding |
| **AFLA_131330** | -2.72 | *nsdC* | $C_2H_2$ |
| **AFLA_087350** | -2.86 | *sltA* | $C_2H_2$ |
| **AFLA_017640** | -2.87 | *rpn4* | $C_2H_2$ |
| **AFLA_029620** | -2.96 | *abaA* | TEA/ATTS |
| **AFLA_137320** | -3.04 | *flbC* | $C_2H_2$ |

| | | | |
|---|---|---|---|
| **AFLA_069460** | -3.11 | *egr2* | $C_2H_2$ |
| **AFLA_069100** | -3.18 | Putative TF | LIM/homeobox |
| **AFLA_082850** | -3.31 | *brlA* | $C_2H_2$ |
| **AFLA_086110** | -3.54 | Putative TF | $C_6$ |
| **AFLA_057480** | -4.22 | Putative TF | Zinc-binding |
| **AFLA_021240** | -4.26 | *glcD gamma* | HLH |
| **AFLA_059960** | -4.33 | *aoiH* | $C_6$ |
| **AFLA_113790** | -5.05 | Putative TF | bZIP |
